# Treatment advances in high-grade gliomas

**DOI:** 10.3389/fonc.2024.1287725

**Published:** 2024-04-10

**Authors:** Xi Chen, Yi Cui, Liqun Zou

**Affiliations:** ^1^ Department of Radiotherapy, Cancer Center, West China Hospital of Sichuan University, Chengdu, China; ^2^ State Key Laboratory of Biotherapy, Sichuan University, Chengdu, China; ^3^ Department of Medical Oncology, Cancer Center, West China Hospital of Sichuan University, Chengdu, China

**Keywords:** high-grade gliomas, glioblastoma, cytotoxic chemotherapy, tumour treating fields, radiotherapy, targeted therapy, immunotherapy

## Abstract

High-grade gliomas (HGG) pose significant challenges in modern tumour therapy due to the distinct biological properties and limitations of the blood-brain barrier. This review discusses recent advancements in HGG treatment, particularly in the context of immunotherapy and cellular therapy. Initially, treatment strategies focus on targeting tumour cells guided by the molecular characteristics of various gliomas, encompassing chemotherapy, radiotherapy and targeted therapy for enhanced precision. Additionally, technological enhancements are augmenting traditional treatment modalities. Furthermore, immunotherapy, emphasising comprehensive tumour management, has gained widespread attention. Immune checkpoint inhibitors, vaccines and CAR-T cells exhibit promising efficacy against recurrent HGG. Moreover, emerging therapies such as tumour treating fields (TTFields) offer additional treatment avenues for patients with HGG. The combination of diverse treatments holds promise for improving the prognosis of HGG, particularly in cases of recurrence.

## 1 Introduction

Gliomas originate from glial cells that differentiate from the neuroectoderm and constitute approximately 80% of primary brain malignancies in adults (1). The 2021 WHO classification integrates histological characteristics and molecular phenotypes to delineate the types and subtypes of central nervous system (CNS) tumours (2). Gliomas are categorised into five types: Adult-type diffuse gliomas, Paediatric-type diffuse low-grade gliomas, Paediatric-type diffuse high-grade gliomas (HGG), Circumscribed astrocytic gliomas and Ependymal tumours. Within these types, tumours are graded according to the CNS WHO Grades. **Table 1** outlines the subtypes of different glioma types that may progress to HGG or have been identified as HGG, along with associated genetic or molecular changes essential for accurate diagnosis.

**Table 1:** Subtypes of different types of high-grade gliomas that may develop into high-grade gliomas and their diagnostic genetic or molecular changes.

Types	Subtypes	Grade	Genetic/Molecular Changes
Adult-type diffuse gliomas	Astrocytoma, IDH-mutant	3, 4	IDH1, IDH2, ATRX, TP53, CDKN2A/B
	Oligodendroglioma, IDH-mutant, and 1p/19q-codeleted	3	IDH1, IDH2, 1p/19q, TERT promoter, CIC, FUBP1, NOTCH1
	Glioblastoma, IDH-wildtype	4	IDH-wildtype, TERT promoter, chromosomes 7/10, EGFR
Pediatric-type diffuse high-grade gliomas	Diffuse midline glioma, H3 K27-altered		H3 K27, TP53, ACVR1, PDGFRA, EGFR, EZHIP
	Diffuse hemispheric glioma, H3 G34-mutant	4	H3 G34, TP53, ATRX
	Diffuse pediatric-type high-grade glioma, H3-wildtype and IDH-wildtype		IDH-wildtype, H3-wildtype, PDGFRA, MYCN, EGFR (methylome)
	Infant-type hemispheric glioma		NTRK family, ALK, ROS, MET
Circumscribed astrocytic gliomas	High-grade astrocytoma with piloid features		BRAF, NF1, ATRX, CDKN2A/B (methylome)

The standard treatment for HGG entails maximal surgical resection, followed by standard fractionated radiotherapy and concurrent and/or adjuvant chemotherapy (1). This approach is considered the most efficacious for HGG and is frequently complemented by targeted therapy, immunotherapy and other comprehensive interventions (2). Glioblastoma (GBM), the most prevalent grade IV WHO glioma in adults, exhibits an exceedingly poor prognosis, with a median overall survival (OS) of 14.6 months under standard therapy and a 5-year survival rate of less than 10% (3, 4). This presents a significant challenge in modern treatment, given that relapse occurs in nearly all cases, with limited treatment options upon recurrence.

Despite a significant increase in the number of cancer treatments approved by the U.S. Food and Drug Administration (FDA) over the past decade, only three new treatments have gained approval for GBM since 2005: Temozolomide (TMZ), Bevacizumab (BVZ) and Tumour Treating Fields (TTFields) (3, 5, 6).

**Figure 1:**
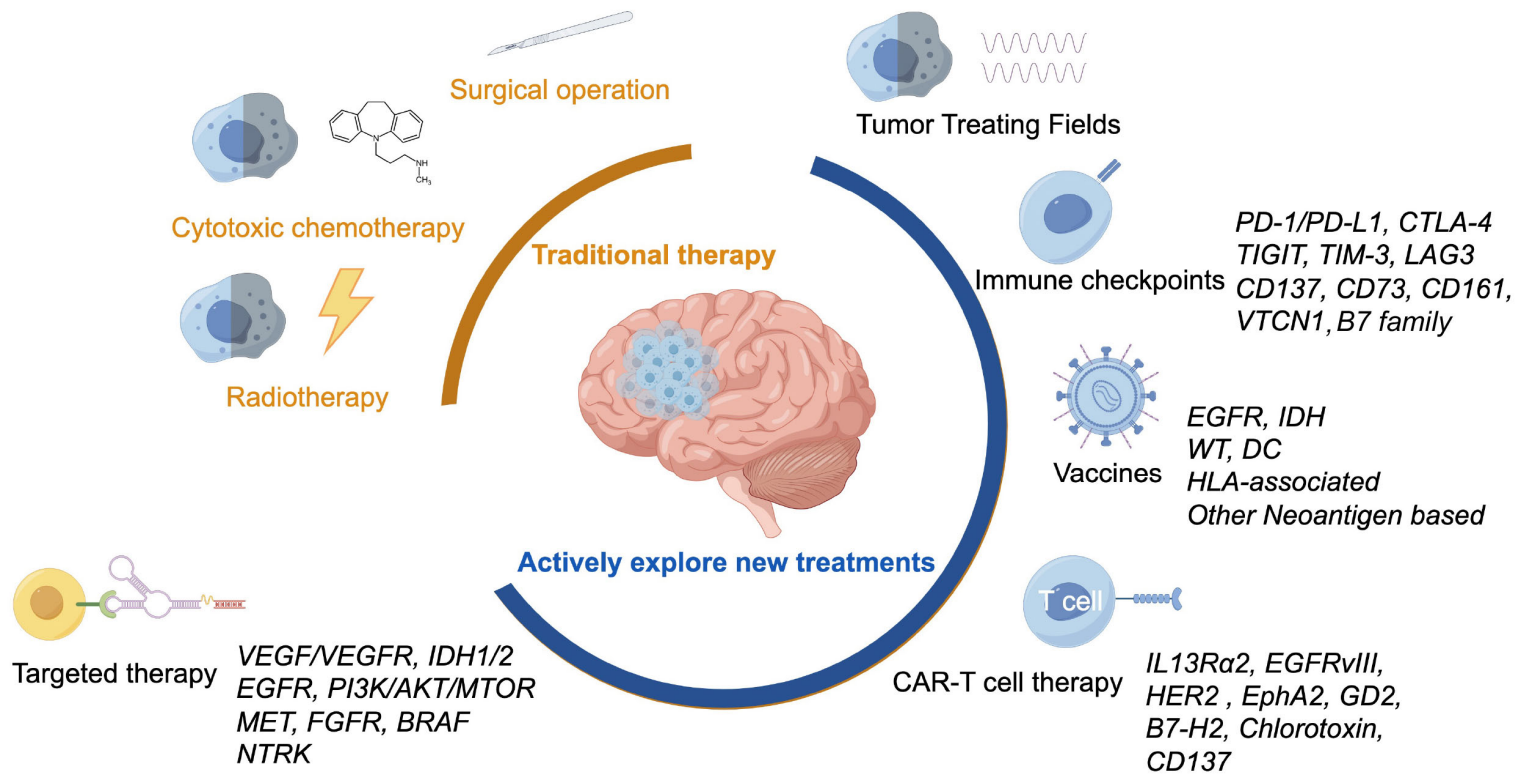
Various treatment methods for glioblastoma, including traditional treatments and actively explored new treatments.

The unique biological complexity of gliomas has prompted researchers to explore novel research avenues that can be translated into clinical practice. This paper systematically reviews recent advancements in HGG treatment, especially glioblastoma, spanning from traditional chemotherapy and radiotherapy to the forefronts of targeted, immune and TTFields therapies, drawing upon the latest literature as shown in **Figure 1**.

## 2 Cytotoxic chemotherapy

Currently, the standard treatment for HGG entails the administration of oral or intravenous TMZ. Patients with GBM who exhibit greater O6-methylguanine-DNA methyltransferase (MGMT) promoter methylation tend to be more responsive to TMZ treatment (7, 8). Evidence suggests that assessing MGMT methylation status can aid in risk stratification and in identifying patients who may benefit from intensive TMZ therapy for other types of HGG (4, 9). A recent large-scale study involving newly diagnosed grade III patients with glioma without 1p19q codeletion indicated that those with MGMT methylation exhibited a higher median OS compared to those without methylation. Furthermore, it is suggested that MGMT methylation correlated with improved OS in patients who received adjuvant chemoradiotherapy or adjuvant radiotherapy, though it did not impact survival in patients who underwent adjuvant chemotherapy or received no adjuvant therapy (10).

The blood-brain barrier (BBB) poses a significant challenge to achieving effective systemic chemotherapy accumulation at the tumour site, resulting in suboptimal treatment efficacy for gliomas. However, convection-enhanced delivery (CED) of chemotherapy can bypass the BBB and directly administer agents into the tumour and surrounding parenchyma through continuous positive-pressure infusion. CED has demonstrated the ability to extensive distribution volumes and deliver a wide range of compounds. Studies in glioma models have shown that the CED of TMZ is safer and more effective than systemic administration. Nevertheless, challenges persist regarding drug distribution limitations and inadequate brain accumulation. To address these issues, researchers have explored modifications to CED technology, including the utilisation of nanoparticles containing an oxaliplatin prodrug and a cationic DNA intercalator. These nanoparticles have shown promise in inhibiting the growth of TMZ-resistant cells in patient-derived xenografts and impeding the progression of TMZ-resistant human GBM in mice, potentially circumventing TMZ resistance. Despite these encouraging results, further investigations are necessary to effectively translate CED technology for CNS tumour treatment into a clinically viable platform.

Liposomes have emerged as a potential strategy to enhance the efficacy of CED by facilitating drug delivery into the brain. One effective approach involves encapsulating TMZ within hydrophilic (PEGylated) liposomes to shield it from hydrolysis and increase drug accumulation in tumour cells. However, an *in vitro* study utilising a TMZ liposome-CED formulation failed to demonstrate clear advantages over conventional drug solutions (11). Conversely, a dual-targeting immuno-liposome encapsulating TMZ (Dual-LP-TMZ) exhibited effectiveness in delivering TMZ to glioblastoma stem cells across the BBB, indicating its potential as a therapeutic option for GBM (7). In summary, utilising liposomal or nanocarrier systems to deliver TMZ or other chemotherapy drugs represents a feasible approach, yet further research is necessary to optimise these delivery systems.

## 3 Tumour treating fields

In recent years, TTFields has emerged as a novel, noninvasive and nontoxic approach to anticancer treatment, challenging traditional modalities. This represents a significant therapeutic advancement in cancer treatment over the past decade.

Several hypotheses have been proposed to explain the mechanism of action of TTFields against cancer. One hypothesis suggests that intracellular elements, such as organelles and macromolecules, are polar and susceptible to external electric fields. Additionally, mitotic cells harbour highly polar and dynamic spindle microtubules (12). TTFields utilise alternating mid-frequency electrical frequencies (200 kHz) and low field strengths (1–3 V/cm) to disrupt the mitotic spindle, impede its normal formation and activate the spindle assembly checkpoint (SAC). This activation leads to the induction of mitotic catastrophe, autophagy and apoptosis in tumour cells (13).

In early 2007, Kirson et al. conducted a preliminary clinical trial to assess the impact of TTFields on 10 patients with relapsed GBM. The study revealed that TTFields more than doubled the time to disease progression and OS compared to the median reported in historical control patients (14). This initial clinical study affirmed the safety and efficacy of TTFields, prompting rapid development in subsequent years. Although the EF-11 phase III study did not demonstrate a significant improvement in the OS rate of relapsed GBM compared to chemotherapy, both treatments were found to be comparable. However, the results of toxicity and quality of life favoured TTFields, leading to FDA approval of TTFields monotherapy for recurrent GBM (15).

The EF-14 study (NCT00916409) enrolled 695 GBM-naive patients who had undergone surgery or biopsy, radiation and TMZ chemotherapy. This study compared TTFields plus maintenance TMZ chemotherapy to TMZ alone. Results indicated a median progression-free survival (PFS) of 6.7 months versus 4.0 months and a median OS of 20.9 months versus 16.0 months, with similar rates of systemic adverse events between the two groups (16). Subgroup analysis revealed that TTFields combined with TMZ improved PFS (6.5 months vs. 3.9 months) and OS (17.4 months vs. 13.7 months) in patients aged ≥65 years with a worse prognosis, compared to TMZ chemotherapy alone (17). Furthermore, in a Korean population analysis, TTFields combined with TMZ showed higher median OS and 1- and 2-year survival rates, comparable to those in the general population data from the EF-14 study (18). Overall, both the population and subgroup analyses demonstrated the efficacy and safety of TTFields in patients with newly diagnosed GBM, leading to FDA approval of TTFields therapy for adults with newly diagnosed GBM.

Regarding safety, skin irritation related to the array was the most common adverse event (AE) associated with TTFields, according to two phase 3 studies, namely EF-11 in relapsed and EF-14 in newly diagnosed GBM. Global post-marketing safety monitoring of TTFields in clinical use in patients with HGG revealed no new safety concerns, with mild-to-moderate and manageable skin reactions related to the array remaining the most common AEs, with an overall incidence not exceeding 38%. The incidence in children, adults and older patients was 37%, 34% and 36%, respectively. Other TTFields-related AEs included warmth under the panel, tingling inductance, or headache, with the incidence of each AE not exceeding 11%. The safety profile was consistent across subgroups, including older adults, indicating feasibility in multiple populations (19).

Despite its efficacy and safety, TTField therapy is often associated with low acceptance and compliance rates. Currently, the therapy involves delivering alternating electric fields to the brain tumour through skin transducer arrays, which can be challenging for patients due to several factors such as hair shaving, frequent array changes, device weight and spare batteries, array visibility, increased sweat rate in warm temperatures, device alarm tone, and orthopaedic issues. These factors may limit patient acceptance of TTFields therapy, highlighting the need to address these challenges and improve patient acceptance and compliance. For instance, a study conducted in Germany showed that only 36% of 30 newly diagnosed patients with GBM accepted TTFields therapy, emphasising the need to improve acceptance rates (20). Therefore, addressing these challenges is imperative to improve patient acceptance and compliance with TTFields therapy.

There is evidence suggesting that TTFields may also modulate immune pathways. T-cells have been observed to maintain viability and function under TTFields treatment, displaying increased IFN-γ secretion, cytotoxic degranulation and PD-1 expression. This suggests that combining TTFields with T-cell-based immunotherapy approaches may be therapeutically beneficial (21, 22). Furthermore, TTFields have been found not only to release and expose molecules associated with cellular damage but also to promote DCs to phagocytose cancer cells, induce DC maturation *in vitro* and recruit immune cells *in vivo*. Combining TTFields with anti-PD-1 therapy has demonstrated a significant reduction in tumour volume and an increase in tumour-infiltrating immune cells in orthotopic lung and colon cancer tumour models (23). TTFields-mediated cell death may trigger anti-tumour immunity and can be effectively combined with anti-PD-1 therapy, providing a dual advantage. The molecular mechanism through which TTFields regulate immunity has been gradually elucidated, with Bezu et al. suggesting that TTFields induce stress responses in the endoplasmic reticulum, evidenced by the phosphorylation of eukaryotic initiation factor α (eIF2α), a characteristic feature of immunogenic cell death (24). eIF2α phosphorylation is associated with the surface exposure of calreticulin in tumour cells, which elicits a favourable anticancer immune response (25, 26). Chen et al. have also investigated TTFields’ role in revitalising the immune response, demonstrating that TTFields promote the production of immunostimulatory pro-inflammatory cytokines and type 1 interferon cytokines in tumour cells via an inflammasome-dependent mechanism, thereby activating the immune system (27). Further examination of these mechanisms of TTFields’ action is warranted.

Preclinical models have indicated that TTFields can reversibly influence BBB permeability by delocalising tight junction proteins such as claudin-5 from the membrane to the cytoplasm (28). This effect facilitates enhanced central delivery of chemotherapy drugs and immune macromolecular drugs. Overall, the tolerable safety profile and prolonged OS associated with TTFields render it an appealing treatment option for patients with brain tumours. Combining TTFields with other therapies can offer significant clinical advantages while minimising additional toxicity. Nevertheless, there is still much to uncover about the mechanisms of TTFields in GBM therapy, necessitating further exploration and optimisation of combination therapies.

## 4 Radiotherapy

Radiotherapy is a viable treatment option for select patients with brain tumours. Advanced radiation technologies, such as intensity modulated radiotherapy (IMRT), volume modulated arc therapy (VMAT), stereotactic radiosurgery (SRS) or stereotactic radiotherapy (SRT), are widely employed techniques. IMRT and VMAT offer enhanced target conformity and better preservation of key tissues, such as the hippocampus and brainstem, thereby potentially reducing late toxicity associated with radiation therapy (29). These advantages underscore the superiority of IMRT and VMAT over three-dimensional conformal radiotherapy (3DCRT) across various brain tumour types. SRS and SRT, whether delivered in hypofractionated or conventional regimens, can effectively slow fractionation and have demonstrated improved survival rates after re-irradiation in recurrent patients with GBM in multiple studies (30, 31). The precise delineation of the target volume and organs at risk (OARs) is paramount in re-irradiation. Routine use of magnetic resonance imaging (MRI) in conjunction with contrast-enhanced T1 and T2-weighted sequences, along with surrounding abnormal FLAIR signal lesions, is recommended for target volume delineation. Evidence suggests that irradiating areas inclusive of those with abnormal FLAIR distribution can improve local tumour control and reduce local tumour progression (32).

However, questions persist regarding the efficacy and potential toxicity of repeat radiotherapy. While primary radiotherapy is generally considered safe and effective, re-irradiation may entail the risk of significant neurotoxicity. A study involving 31 patients with HGG, 81% of whom had GBM, revealed limited efficacy of re-irradiation, with a median OS of 7.0 months and a median PFS of 2.8 months. Among patients achieving disease control, 43% experienced late toxicity in the form of radiation necrosis and irreversible white matter changes, which, albeit manageable, underscore the importance of careful consideration (33). Therefore, re-irradiation should be judiciously employed, preferably restricted to small-volume recurrences, especially in regions unexposed to radiation for at least six months post-completion of previous radiotherapy. Given the treatment plan’s complexity, re-irradiation should be subject to real-time review or phased therapy, whenever feasible.

The efficacy of combining re-irradiation with systemic therapy for recurrent HGG remains a matter of debate. Some studies have indicated that combining re-irradiation with bevacizumab as salvage therapy can lead to prolonged survival with minimal toxicity (34, 35). However, other studies have reported that this combination solely improves PFS without a corresponding increase in OS (36). Exploring the combination of re-irradiation with bevacizumab in patients with HGG having small, confined or IDH-mutated lesions may be worthwhile. Furthermore, concomitant and/or adjuvant TMZ therapy alongside radiation has shown improved OS and PFS, predominantly in MGMT-methylated tumours (37).

## 5 Targeted therapy

Targeted therapy, a treatment method aimed at precisely targeting cancer cells, exerts its effect by targeting specific genes and proteins within tumour cells.

### 5.1 Targeting growth factor receptors signalling

Advancements in understanding the molecular pathways associated with glioblastoma have facilitated the identification of tumour biomarkers, paving the way for the development of drugs targeting tumour cells and the tumour microenvironment. Particularly in the tyrosine kinase receptor pathway, several targets have been identified, including epidermal growth factor receptor (EGFR), PI3K/AKT/mTOR pathway, MET, Fibroblast growth factor receptor (FGFR), BRAF mutation, Neurotrophic tyrosine receptor kinases (NTRK) and Vascular endothelial growth factor (VEGF), among other. These targets have been elaborated upon in several reviews (38).

### 5.2 Targeting tumour angiogenesis

Gliomas are characterised by a dense network of blood vessels and high expression levels of VEGF, which promotes the formation of new blood vessels (angiogenesis). In the United States, two independent studies resulted in the FDA approval of bevacizumab for the treatment of relapsed GBM in 2009 (5, 39). Bevacizumab operates by binding to and inhibiting vascular endothelial growth factor A (VEGF-A). As additional clinical trials are conducted and results are updated, several studies have provided compelling evidence regarding both the benefits and limitations of bevacizumab’s antiangiogenic therapy in patients with HGG.

An analysis of 11 studies conducted between 2014 and 2017, involving patients with newly diagnosed or relapsed HGG treated with neoadjuvant or adjuvant therapy, revealed that bevacizumab antivascular therapy improved PFS but not OS (40). Similarly, a review of 52 studies related to GBM conducted between 2000 and 2016 supported the efficacy of bevacizumab, either alone or in combination with chemotherapy, in extending PFS and OS in relapsed cases, but not in newly diagnosed cases (41). Thus, the use of antiangiogenic therapy in patients with newly diagnosed GBM is still yet fully supported by evidence.

Furthermore, the superiority of antiangiogenic therapy over chemotherapy in recurrent GBM is still under investigation, considering potential AEs, such as hypertension, proteinuria, slow wound healing and thromboembolic events, which must be carefully weighed against the patient’s quality of life. In the reviewed studies, an average of one AE occurred per patient, with 74% of them classified as grade 3 or higher toxicity (41).

A novel antivascular targeting drug, apatinib, has emerged as a potential therapeutic option for HGG. Apatinib functions by inhibiting the phosphorylation of tyrosine residues in the intracellular domain of VEGF receptor 2 (VEGFR2), thereby inhibiting the downstream biological effects of VEGF (42). It can penetrate the BBB, suppress glioma cell growth and metastasis, induce ferroptosis by inhibiting the activation of the nuclear factor erythrocyte-related factor 2 (Nrf2)/VEGFR2 pathway and result in the loss of tumour cell viability (43).

*In vitro* studies have demonstrated that apatinib can enhance the anti-tumour activity of TMZ (44), while *in vivo* experiments revealed that the combination of apatinib and TMZ can improve disease control efficacy in patients with recurrent HGG, particularly those with poor Karnofsky performance (45). Nevertheless, further investigations are required to validate these findings.

### 5.3 Targeting mutation

Reports suggest that approximately 91% of GBMs expressing wild-type isocitrate dehydrogenase (IDH) have a median OS of 1.2 years, while 9% of patients with IDH mutations have a median OS of 3.6 years (46). IDH1/2 are metabolic enzymes involved in the conversion of isocitrate to α-ketoglutarate (α-KG) in the tricarboxylic acid cycle (47). Somatic mutations, such as R132H in IDH1 and R140Q or R172H in IDH2, activate a novel pathway that converts α-KG to D-2-hydroxyglutarate (D-2-HG), a competitive antagonist of α-KG. This inhibits the activity of α-KG-dependent dioxygenases, including enzymes involved in histone and DNA demethylation, contributing to glioma progression (48, 49).

Mutant IDH1/2 exhibits distinct enzymatic activity compared to wild-type IDH1/2, and specific inhibitors targeting the mutant enzyme should not interfere with wild-type enzyme activity. D-2-HG, the product of mutant IDH1, has no physiological function, and inhibiting its synthesis should be safe. These distinguishing features make mutant IDH1/2 an attractive target for IDH-mutant tumour therapy, particularly the development of small molecule inhibitors of mutant IDH enzymes. **Table 2** summarizes some novel inhibitors targeting IDH1 mutation.

**Table 2:** Clinical studies of IDH inhibitors for HGG in recent years.

Drug	NCT	Phase	n	Target	Mode of Administration	Common Adverse Events	Median Progression-free Survival	Reference	Year
DS-1001	NCT03030066	I	47	brain-penetrant mutant IDH1R132X selective inhibitor.	125-1400 orally mg twice daily.	Skin hyperpigmentation, diarrhea, pruritus, alopecia, arthralgia, nausea, headache, rash, and dry skin.	10.4 months for patients with enhancing glioma; not reach for patients with nonenhancing glioma.	(50)	2023
Olutasidenib (FT-2102)	NCT03684811	Ib/II	26	brain-penetrant IDH1 selective inhibitor.	150 mg orally twice daily; in 28-day cycles until progression or unacceptable toxicity.	dose-limiting toxicities (DLTs).	15.1 months.	(51)	2023
Vorasidenib (AG-881)	NCT02481154	I	93	brain-penetrant, IDH1 and IDH2 dual inhibitor.	25-300 mg orally once daily in glioma; 25-400 mg orally once daily in nonglioma; in 28-day cycles until progression or unacceptable toxicity.	Dose-limiting toxicities of elevated transaminases occurred at doses ≥100 mg.	3.6 months for patients with enhancing glioma; 36.8 months for patients with nonenhancing glioma.	(52)	2021

One such inhibitor is the IDH1 ubiquitous protein inhibitor, BAY 1436032, which possesses distinct R132 codon mutations that significantly decrease 2-HG levels in cells harbouring the corresponding IDH1 mutations but have no effect on cells lacking IDH mutations. BAY 1436032 demonstrated no toxicity *in vitro* or *in vivo* and significantly prolonged the survival of mice implanted with IDH1-R132H-mutated human astrocytoma (53). Another inhibitor is MRK-A, a brain-penetrating IDH1 mutant-specific inhibitor with the potential to impair 2-HG synthesis. MRK-A offers significant survival benefits *in vivo*, despite having minimal effect on IDH1-mutant glioma cell proliferation *in vitro* (54).

A recent study utilising proteomics classified wild-type IDH GBM into two distinct proteome subtypes with stable characteristics. Cohort 1 was the only group exhibiting EGFRvIII and PIK3CA mutations, while other GBM-associated mutations such as TP53, NF1, PTEN and RB1 were more evenly distributed between the two subtypes, including non-EGFRvIII mutations (55). Thus, these findings could aid in determining GBM prognosis and developing more targeted treatment approaches.

## 6 Immunotherapy

While the brain was traditionally considered immune-privileged, recent research suggests that the immune system can interact with CNS cells due to disruptions in the BBB and the presence of lymphatic outflow channels during inflammation or malignancy (56–58). Various factors such as traumatic brain injury, autoimmunity, metabolic toxicity or misfolded protein accumulation can induce inflammation in the CNS, facilitating the entry of peripheral immune cells across the BBB (59). The discovery of functional lymphatic vessels lining the dural sinuses in rodents in 2015, wherein they resemble traditional lymphatic pathways and run parallel to the dual venous sinuses (60), provides a convention. conventional route for immune cells to traffic across the CNS, reshaping our understanding of the immune environment of brain tumours.

Upon the release and detection of endogenous danger molecules, peripheral immune cells swiftly infiltrate the CNS by crossing the BBB, triggering robust inflammatory responses. This phenomenon is speculated to be vital for effective immunotherapy targeting brain tumours (61). Recent studies have reinforced this hypothesis, revealing mechanisms such as tumour-infiltrating lymphocyte (TIL) anergy, recruitment of immunosuppressive regulatory T-cells (Tregs) and activation of immunological checkpoints by GBM (62, 63).

### 6.1 Immune checkpoint inhibitors

Since the FDA approved immune checkpoint inhibitors (ICIs) targeting CTLA4 and PD-1 for melanoma treatment, ICIs have revolutionised tumour therapy (64). Concurrently, PD-1 and PD-L1 blockade therapy has rapidly gained traction for treating various solid tumours, including lung cancer, breast cancer and kidney cancer, among others (65). However, clinical evidence supporting PD-1 blockade therapy in glioma remains insufficient. Hence, the application of immunotherapy in HGG has sparked significant interest, with PD-1/PD-L1-based ICIs emerging as a prominent area of research.

#### 6.1.1 PD-1/PD-L1

##### 6.1.1.1 Expression of PD-L1 in glioma

Numerous studies have explored the relationship between PD-L1 expression levels and prognosis in patients with glioma. While some studies suggest no association between PD-L1 expression and glioma prognosis (66, 67), the majority of research (68–70) confirms a correlation between high PD-L1 expression and poor prognosis.

A study incorporating data from 1052 patients from the China Gliomas Genome Atlas (CGGA) and 976 patients from the Cancer Genome Atlas (TCGA) revealed that patients with concomitant gliomas and high PD-L1 mRNA expression exhibited significantly shorter OS (68). Monoclonal antibodies targeting PD-1 have been investigated in multiple relapsed GBM.

Another study highlighted the concentration of the PDCD1 gene, which encodes PD-1, in IDH-wild-type gliomas. Genes associated with PDCD1 are implicated in the inflammatory immune response and T-cell-mediated immune response in glioma (71). The expression of PD-L1 in gliomas has been well established.

Furthermore, Berghoff et al. reported PD-L1 expression in 88% of initial diagnoses and 72.2% in recurrent GBM specimens (72). Nduom et al. found that 38% of 94 patients with GBM had at least 5% or more tumour cells positive for PD-L1 expression (73). Similarly, Ding et al. identified a strong correlation between tumour grade and PD-L1 expression in 41.7% of tumour cells from 120 patients with glioma (74).

The genotype of gliomas has been linked to PD-L1 expression, with significantly higher PD-L1 expression observed in wild-type IDH gliomas compared to IDH-mutant gliomas across various stages of gliomas (68, 69). This difference could be attributed to the increased methylation of the mutant PD-L1 promoter (70).

##### 6.1.1.2 Clinical research of PD-1/PD-L1 ICIs in HGG

In a phase II study (NCT02337491) involving 80 patients with recurrent GBM who had not previously received bevacizumab, the PD-1 inhibitor pembrolizumab combined with bevacizumab demonstrated a 6-month PFS of 26.0%, a median OS of 8.8 months and an objective response rate (ORR) of 20%. When pembrolizumab was administered alone, the 6-month PFS was 6.7%, the median OS was 10.3 months and the ORR was 0%. While pembrolizumab, either alone or in combination with bevacizumab, was well tolerated, it provided limited benefit (75).

Two studies focusing on HGG also showed limited survival benefit in patients treated with PD-1 inhibitors pembrolizumab or nivolumab alone or in combination with bevacizumab salvage therapy (76, 77). CheckMate 143 (NCT02017717), a large phase III randomised clinical trial evaluating PD-1 pathway inhibitors in GBM, enrolled 369 patients and compared the PD-1 monoclonal antibody nivolumab monotherapy with bevacizumab in the treatment of first relapsed disease. Although nivolumab did not prolong OS in patients with relapsed GBM, a small proportion (8%) of patients exhibited a longer response duration to nivolumab than to bevacizumab (11.1 months vs. 5.3 months) (78).

The limited efficacy of monoclonal antibody PD-1 in treating recurrent HGG has led to its exploration in the neoadjuvant treatment model, showing promise.

In a randomised, multicenter clinical trial of neoadjuvant therapy with pembrolizumab in 35 patients with recurrent, resectable GBM, continuing the drug after surgery significantly prolonged OS by 6.2 months and PFS by 0.9 months compared to patients treated with pembrolizumab only after surgery. The study demonstrated that neoadjuvant anti-PD-1 immunotherapy not only directly affected tumour cells but also acted through a systemic immune response. This was evidenced by T-cell blockade, increased interferon-γ-related gene expression, local PD-L1 induction in the tumour microenvironment, increased T-cell clonal expansion, decreased PD-1 expression in peripheral blood T-cells and monocyte populations (79).

In another phase II clinical trial (NCT02550249), 27 recurrent cases and 3 treatment-naïve patients with GBM received nivolumab before surgery and continued treatment postoperatively until disease progression or unacceptable toxicity. No clinical benefit was found in relapsed patients, but two treatment-naïve patients were still alive after 33 and 28 months. This study also found that neoadjuvant PD-1 monoclonal antibody therapy enhanced chemokine transcript expressions, increased immune cell infiltration, enhanced TCR clone diversity of tumour-infiltrating T lymphocytes and enhanced local immune regulation (80).

##### 6.1.1.3 Effect of immune microenvironment on immunotherapy of HGG

The aforementioned studies suggest that PD-1 blockade induces T-cell activation and infiltration in the tumour microenvironment of GBM, altering the tumour immune microenvironment. They also highlight the role of the tumour immune system in tumour immunotherapy.

Changes in immune cell infiltration before and after neoadjuvant PD-1 monoclonal antibody therapy indicate its potential to induce the activation of T-cells and type 1 dendritic cells (cDC1s). However, after anti-PD-1 therapy, macrophages and monocytes still constitute the majority of infiltrating immune cells and exhibit poor efficacy against immunosuppressive tumour-associated macrophages in recurrent GBM (81).

The primary clinical results of ICIs are summarised in **Table 3**. Overall, preliminary clinical trials of ICIs in glioma have yielded limited outcomes in primary and recurrent HGG compared to most solid tumours. Firstly, patients with GBM experience decreased levels of circulating CD4+ and CD8+ lymphocytes and widespread immunological dysfunction, exacerbated by lymphocyte-depleting therapies like chemotherapy (82, 83). Such therapies impact PD-1 antibody and PD-1 receptor interactions in lymphocytes. Secondly, drug permeability is limited in the presence of the BBB due to CNS pathophysiology, with compounds larger than 400-600 Da unable to permeate. This is unfavourable for ICI macromolecules (84). Pembrolizumab has a molecular weight of 149 kDa and nivolumab has a molecular weight of 145 kDa, suggesting that PD-1/PD-L1 axis-mediated antibody inhibition occurs outside the tumour. Effector T-cells in peripheral lymphoid tissue activate tumour-associated antigens, which then enter the tumour microenvironment and interact with anti-PD-1 antibodies (85).

**Table 3:** Results of major clinical studies of PD-1/PD-L1 inhibitors for HGG.

Trial Number	Trial phase	Subtype	Experimental arm	Reference arm	n	Experimental arm Survival	Reference arm Survival	Reference	Year
NCT02337491	II	Relapsed-GBM	Pembro+ Beva	Pembro	80	6 m-PFS 26% mOS 8.8 m ORR 20%	6 m-PFS 6.7% mOS 10.3 m ORR 0%	(75)	2020
Retrospective	—	Refractory -HGG	Pembro, Pembro+ Beva, Pembro+ Beva+TMZ	—	24	PR 8.3%, SD 20.8% mPFS 1.4 m mOS 4 m	—	(76)	2017
Retrospective	—	Relapsed-HGG	Pembro/Nivo+ Beva	—	31	mPFS 3.2 m mOS 6.6 m	—	(77)	2018
NCT02017717	III	Relapsed -GBM	Nivo	Beva	369	mOS 9.8 m ORR 7.8%	mOS 10 m ORR 23.1%	(78)	2020
—	—	Relapsed-GBM	Neoadjuvant Pembro + Adjuvant Pembro	Adjuvant Pembro	35	mPFS 3.3 m mOS 13.7 m	mPFS 2.4 m mOS 7.5 m	(79)	2019
NCT02550249	II	Relapsed/Newly diagnosed GBM	Neoadjuvant Nivo+ Adjuvant Nivo	—	30	mPFS 4.1 m mOS 7.3 m	—	(80)	2019

HGG, high-grade gliomas; GBM, glioblastoma; ORR, overall response rate; PR, partial response; SD, stable disease; mOS, median overall survival; mPFS, median progression-free survival; Pembro, Pembrolizumab; Beva, Bevacizumab; Nivo, Nivolumab; TMZ, temozolomide; m, month.

#### 6.1.2 Advances in other immune checkpoints

##### 6.1.2.1 CTLA-4

Cytotoxic T-lymphocyte-associated antigen 4 (CTLA4, CD152) is a co-inhibitory molecule (86) Chimeric fusion proteins consisting of the ectodomain of CTLA-4 can bind to B7-1 and B7-2, blocking T-cell activation (87). Statistical analysis based on clinical data has shown that CTLA-4 expression is higher in patients with higher grade, isocitrate dehydrogenase (IDH)-wild-type and mesenchymal-molecular subtype gliomas compared to patients with lower grade, IDH-mutant and other molecular subtype gliomas (88). This suggests that the expression pattern and clinical characteristics of CTLA-4 in glioma are related to tumour severity (89). Ipilimumab, an immune checkpoint inhibitor targeting CTLA-4, when combined with temozolomide was reported to not improve PFS or OS in patients with glioblastoma (90, 91). Another clinical trial reported that the intracerebral injection of ipilimumab plus the PD-1 blocking mAb nivolumab is feasible, safe and results in encouraging long-term OS (92). Therefore, combining additional immune checkpoint-blocking monoclonal antibodies may be a promising approach to further improve outcomes for patients with recurrent glioblastoma.

##### 6.1.2.2 TIM-3

T-cell immunoglobulin and mucin-domain containing-3 (TIM-3) is emerging as an important immune checkpoint molecule presents in various immune cells, such as T-cells, B cells, natural killer (NK) cells, dendritic cells, monocytes, macrophages and NK, and tumour cells (93). Intratumoral TIM-3 expression by CD4+ and CD8+ T-cells was found to be higher in GBM compared with low-grade glioma, suggesting an association with glioma severity (94). Research has shown that triple therapy with anti-TIM-3 antibody, anti-PD-1 antibody and stereotactic radiosurgery (SRS) can achieve 100% OS and increase immune cell infiltration, immune cell activity and memory performance (95). A trial is currently investigating anti-TIM-3 in combination with anti-PD-1 and SRS for the treatment of recurrent GBM (NCT03961971). Additionally, an ongoing open-label, multicenter, nonrandomised phase 1 and 2 clinical trial is evaluating various combinations of an investigational anti-TIM-3 mAb Bgb-A425 with the anti-PD-1 mAb tislelizumab in advanced solid tumours (96).

##### 6.1.2.3 LAG-3

Lymphocyte-activating gene-3 (LAG-3) is an immunoglobulin expressed on various immune cells (NK cells, DC cells, T-cells and B cells) (97, 98) and plays an inhibitory role in T-cell signalling, proliferation and cytokine secretion (99). LAG-3 expression has been observed in human glioblastoma samples and its inhibition has shown efficacy against glioblastoma in preclinical studies. Moreover, it can be used in combination with other immune checkpoint inhibitors (100). Another study reported that TILs expressed LAG-3, which plays a crucial role in the tumour microenvironment (101). Clinical trials have confirmed the safety of combining anti-LAG-3 and anti-PD-1 treatments in glioblastoma (102). Further research should focus on understanding the role of LAG-3 in the tumour microenvironment and investigating the potential of anti-LAG-3 mAb in glioblastoma.

##### 6.1.2.4 CD73

CD73 (ecto-5’-nucleotidase) is an ecto-nucleotidase that dephosphorylates AMP to form adenosine. The activation of adenosine signalling pathways in immune cells leads to the inhibition of effector functions (103). In the tumour microenvironment, adenosine promotes immune suppression through negative feedback signals and is considered an important mechanism for cancer cell immune evasion (104). Glioma-derived CD73 contributes to immune suppression (105). AK119 is an anti-CD73 monoclonal antibody (106). An ongoing phase 1 study (NCT04572152) is evaluating the safety, anti-tumour activity and pharmacokinetics of AK119 in combination with an anti-PD-1/CTLA-4 bispecific antibody in advanced solid tumours (106).

##### 6.1.2.5 CD137

CD137, also known as 4-1BB, is one of the tumour necrosis factor (TNF) receptor family targets (107). It serves as a co-stimulating molecule and regulates various immune cells, including CD4+ cells, CD8+ cells, NK cells, dendritic cells (DC) and regulatory T-cells (Treg) (108). In an *in vitro* model of human glioma, enhanced tumour immune responses were observed when the antibody against CD137 was used (109). Additionally, Puigdelloses et al. constructed Delta-24-ACT, a novel OV armed with 4-1BB ligand (4-1bbL) (110). In GBM murine models, Delta-24-ACT elicited a more potent anti-tumour effect with longer median survival and a higher percentage of long-term survivors (110). However, a recent phase 1 trial study (NCT02658981) involving the anti-CD137 antibody treatment arm was closed. Further clinical trials are needed to evaluate the efficacy of anti-CD137 antibody treatment.

##### 6.1.2.6 TIGIT

T-cell immunoreceptor with immunoglobulin and ITIM domain (TIGIT) is a co-inhibitory molecule expressed on T-cells, including tumour-infiltrating T-cells, Treg, memory subsets and NK cells (111, 112). Preclinical studies have demonstrated that antibodies blocking TIGIT plus PD-L1 or TIM-3 simultaneously can specifically enhance the effector function of CD8+ T-cells, leading to significant anti-tumour immune response (112, 113). Furthermore, TIGIT expression was found to be upregulated in CD8+ T-cells at tumour sites in patients with GBM compared with healthy controls (114). Clinical studies have also demonstrated increased expression of TIGIT on TILs in GBM patient samples (115). Similarly, in a mouse model of GBM, anti-TIGIT combined with anti-PD-1 improved survival compared with monotherapy (115). Another study analysed the TCGA transcriptome database and identified PD1 and TIGIT as preferred targets for GBM immunotherapy. Furthermore, they also reported that the dual blockade of PD1 and TIGIT improved survival and increased CD8+ TIL accumulation and function compared with either drug alone in a murine GBM model (116). Therefore, TIGIT represents a promising target for immunotherapy in patients with GBM, and ongoing clinical trials are evaluating anti-TIGIT combined with anti-PD-1 antibody treatment for recurrent GBM (NCT04656535).

##### 6.1.2.7 B7 family

The B7 family, including B7-1 (CD80), B7-2 (CD86), B7-H1 (PD-L1), B7-DC (CD273, PD-L2), B7-H2 (CD275), B7-H3 (CD276), B7-H4 (VTCN1), B7-H5 (VISTA), B7-H6 (NCR3LG1) and B7-H7 (HHLA2), constitutes a large immune checkpoint family (117). In addition to the classic immune checkpoint PD-L1, B7-H3, B7-H4, B7-H5 and B7-H6 have co-suppressive or costimulatory functions on the immune system (118).

##### 6.1.2.7.1 B7-H3

Initially reported to positively regulate T-cell proliferation (119), subsequent studies found that B7-H3 has an inhibitory effect on cytotoxic T-cells while negatively regulating pro-inflammatory Th1 cells (120). Based on the CGGA and TCGA projects, B7-H3 has been found to be upregulated in higher grade gliomas compared to lower grade gliomas (121). Furthermore, a recent multi-omics analysis reported high B7-H3 expression in multiple cancer types and correlated this upregulation with poorer survival and prognosis (122). Additionally, B7-H3 expression was found to be significantly elevated in GBM compared with normal controls (122). Nonetheless, further preclinical studies and clinical data are needed to elucidate the role of B7-H3 in glioma.

##### 6.1.2.7.2 B7-H4

B7-H4, also known as VTCN1, is an inhibitory molecule expressed on APCs that inhibits T-cells and promotes immune escape (123). Studies have found that B7-H4 expression in tumours is related to the prognosis of human glioblastoma and is directly related to the degree of malignancy (124). B7-H4 activation on macrophages/microglia in the glioma microenvironment is an important immunosuppressive process that prevents effective T-cell immune responses (124). B7-H4 expression was reported to be increased in gliomas with low PD-L1 expression, suggesting potential compensatory immune checkpoint mechanisms in gliomas (125).

##### 6.1.2.7.3 B7-H5

B7-H5, also known as VISTA, is the V-domain Ig suppressor of T-cell activation (VISTA). Within the lymphocyte compartment, VISTA is highly expressed on naive CD4+ and Foxp3+ Regulatory T cells (126). Ghouzlani et al. utilised TCGA data to investigate VISTA expression of 667 patients with glioma using RNA-seq data (127). In this study, VISTA was found to be highly expressed in HGG and associated with poor OS (127). This suggests that B7-H5 may be involved in glioma progression and could become a potential therapeutic target, especially in advanced gliomas.

##### 6.1.2.7.4 B7-H6

B7-H6, also known as natural cytotoxicity triggering receptor 3 (NCR3LG1), plays a crucial role in NK cells and mediated immune responses (128). In U87 and U251 glioma cells, the knockdown of B7-H6 significantly inhibited cell proliferation, migration and invasion but increased apoptosis and enhanced cell cycle arrest (129). Similarly, B7-H6 was found to be expressed in glioma cells and tissues isolated from patients with glioma (130). However, there are currently no ongoing or finished clinical/preclinical trials targeting B7-H6 in glioma or glioblastoma, warranting further research to explore its therapeutic potential. The clinical results mentioned above are summarized in **Table 4**. In summary, there are several types of immune checkpoints besides PD-1/PD-L1, all of which show potential for treating glioblastoma, although more exciting clinical results are needed.

**Table 4:** Results of major clinical studies of other immune checkpoint inhibitors for glioblastoma.

Trial Number	Trial phase	Subtype	Experimental arm	Reference arm	nn	Experimental arm Survival	Reference arm Survival	Reference	Year
ISRCTN84434175	II	Recently diagnosed glioblastoma	ipilimumab + TMZ,	TMZ	119	18 m-PFS 22% mPFS 10.9 m 18 m-OS 53% mOS 22.7 m	18 m-PFS 43% mPFS 12.5 m 18 m-OS 64% mOS 26.4 m	(90, 91)	2020
NCT03233152	I	Recurrent glioblastoma	ipilimumab, ipilimumab plus Nivo	Nivo	27	mPFS 11.7 w mOS 38 w	—	(92)	2021
NCT03493932	I	Recurrent glioblastoma	BMS-986016 + Nivo	—	10	—	—	(102)	2019

mPFS, median progression-free survival; mOS, median overall survival; 18 m-OS, 18 months median overall survival; 18 m-PFS, 18 months median progression-free survival.

### 6.2 Vaccines

Cancer vaccines represent a promising avenue of immunotherapy aimed at eliciting a long-lasting immune response to eliminate tumour cells. The majority of peptide vaccines under investigation for glioma aim to stimulate CD8+ T-cells or CD4+ T helper cells to target tumour-associated antigens (TAAs) or tumour specific antigens (TSAs). CD8+ T-cells, in particular, can recognise human leukocyte antigen (HLA) peptide complexes and generate long-lasting memory cytotoxic T lymphocyte (CTL) responses against antigen-presenting cells (131). Hence, the selection of appropriate tumour antigens to induce specific cytotoxicity against tumour cells is crucial in the development of therapeutic cancer vaccines. In **Table 5**, we have summarized recent clinical studies of vaccine therapy in GBM.

**Table 5:** Results of major clinical studies of vaccines for glioblastoma.

Trial Number	Trial phase	Subtype	Experimental arm	Reference arm	n	Experimental arm Survival	Reference arm Survival	Reference	Year
NCT01480479	III	Newly diagnosed glioblastoma	TMZ + rindopepimut	TMZ + keyhole limpet hemocyanin	745	mOS 20.1 m	mOS 20.0 m	(132)	2017
NCT01498328	II	Relapsed EGFRvIII- Expressing Glioblastoma	Bevacizumab + rindopepimut	Bevacizumab + keyhole limpet hemocyanin	73	6 m-PFS 28% ORR 30%	6 m-PFS 16% ORR 18%	(133)	2020
NCT02454634	I	High grade gliomas	IDH1-vac	—	39	Three-year progression-free 0.63 Death-free rates 0.84	—	(134)	2021
—	II	Recurrent glioblastoma multiforme	WT1 peptide	—	21	mPFS 20.0 w 6 m-PFS 33.3%	—	(135)	2008
UMIN000003506	I	Recurrent malignant glioma	vaccine of WT1	—	14	mOS 24.7w 1-year OS 36%	—	(136)	2019
NCT 01280552	II	Newly diagnosed glioblastoma	ICT-107	—	124	mPFS 2.2 m	—	(137)	2019
NCT01222221	I	Newly diagnosed glioblastoma	IMA950 + GM-CSF	—	45	6 m-PFS 74% 9 m-PFS 31%	—	(138)	2016
NCT02287428	I/Ib	Newly diagnosed glioblastoma	Personalized NeoAntigen Vaccine	—	10	mPFS 7.6 m mOS 16.8 m	—	(139)	2019

ORR, overall response rate; mPFS, median progression-free survival; mOS, median overall survival; 6/9 m-PFS, 6/9 months median progression-free survival.

#### 6.2.1 Vaccines of EGFR

Approximately 40% of newly diagnosed GBMs exhibit EGFR gene amplification, with nearly half of these cases featuring persistently active and oncogenic epidermal growth factor receptor variant III (EGFRvIII) (140, 141). EGFRvIII introduces a novel glycine residue at the junction of exons 1 and 8, creating a unique tumour neoantigen with immunogenicity in humans (142). Therefore, vaccine therapies targeting EGFRvIII have been developed for glioma.

Rindopepimut (CDX-110), the most well-known peptide vaccine targeting EGFRvIII, was first designed in the late 1990s. It has demonstrated the ability to induce cytotoxic T-cell responses and exhibited promising preclinical efficacy in mouse brain tumour models (143). However, in a large-scale randomised, double-blind, phase 3 trial (NCT01480479) investigating the addition of rindopepimut to standard chemotherapy in newly diagnosed patients with glioblastoma, the inclusion of rindopepimut failed to improve survival outcomes. However, rindopepimut displayed favourable tolerability and mitigated the risk of seizure, epilepsy, brain oedema and other adverse effects (132). This prompted a careful review of the design, leading to the refinement of treatment strategies. One such strategy is enhanced proteasome processing, such as the EGFRvIII tyrosine substitution vaccination, which increased median survival in an intracranial glioma model (144).

In a double-blind randomised phase II trial (NCT01498328) involving 73 patients with relapsed EGFRvIII-positive glioblastoma, the addition of rindopepimut to standard bevacizumab treatment conferred a significant survival advantage. Patients who received combination therapy experienced higher 6-month Progression-Free Survival (PFS6), ORR and median duration of response (133). This study underscores the potential clinical utility of targeting EGFRvIII; however, validation from larger trails is warranted to ascertain the heterogeneity of combined drug responses among patients.

#### 6.2.2 Vaccines targeting IDH

The IDH1 mutation, notably affecting codon 132 and encoding IDH1-R132H, presents a shared clonal neoepitope in the class II major histocompatibility complex (MHC) (145, 146). Preclinical data suggests that IDH1-R132H-specific polypeptide vaccines can induce specific therapeutic T helper cell responses and exhibit efficacy against IDH1-R132H-positive tumours in isogenic MHC human mice (145, 147–149).

Taking advantage of the potential positive immune interaction between standard care and vaccination, a multi-centre phase 1 clinical study involving 33 newly diagnosed WHO grade 3 and 4 IDH1-R132H-positive astrocytomas (NCT02454634) demonstrated favourable tolerability of IDH1-R132H-specific polypeptide vaccines. The study reported that vaccine-related AEs were limited to level 1. Notably, up to 93.3% of patients observed a vaccine-induced immune response in multiple MHC alleles, with a two-year PFS rate of 0.82 and a higher rate of survival than those who did not have the immune response (134).

Further investigations have revealed that IDH1 can selectively bind CD8 dimers and enhance immunotherapy effects by augmenting T-cell responsiveness to multiple tumour antigens. However, mutant IDH1R132H exhibits impaired sialidase activity and delayed killing in glioma cells (150), underscoring the complexity of IDH1-mediated immunotherapy in glioma tumours.

#### 6.2.3 Vaccines targeting Wilms’ tumour gene 1

Another known TAA is the product of the Wilms’ tumour gene 1 (WT1) gene. The WT1 gene, encoding a zinc finger transcription factor, was identified as the gene responsible for Wilms tumour. Notably, WT1 plays a role in cell proliferation, differentiation, apoptosis and organ development (151). It is also reported to be present in many solid tumours, including gliomas.

A phase 2 clinical trial evaluated the clinical response of patients with relapsed GBM to peptide immunotherapy targeting the WT1 gene product. Focal erythema appeared only at the injection site of the WT1 vaccine, with a disease control rate of 57.1%, median PFS of 20.0 weeks and PFS6 rate of 33.3%. This study demonstrated acceptable safety profiles and clinical responses of the WT1 vaccines for patients with recurrent GBM (135).

Cocktail treatments of WT1 HLA class I and II vaccinations have shown promising safety profiles and clinical responses in patients with recurrent malignant gliomas. All 14 patients in that study exhibited grade I level of skin disorders at the injection sites. Six of the 14 patients had stable disease at 6 weeks. Overall, median OS was 24.7 weeks and 1-year OS rates were 36% (136). Therefore, these encouraging outcomes underscore the potential of the WTI vaccine as a therapeutic option for patients with recurrent malignant glioma, warranting further investigation in larger clinical studies.

#### 6.2.4 Dendritic cell-based vaccine

DCs, functioning as APCs, are sensitised with TSAs and then administered as a vaccine to stimulate T-cells capable of mounting an anti-tumour response. Multiple small-scale clinical trials have consistently demonstrated the overall safety and provided indirect evidence of clinical efficacy for DC vaccines employing active-specific immunisation strategies targeting the deleterious course of patients with GBM, albeit utilising different non-standardised DC vaccine products (152, 153).

A phase II trial of DC vaccine ICT-107 focused on HLA-A1+ and/or -A2+ GBM patients who underwent surgery with a residual tumour not exceeding 1 cm^3^, followed by radiotherapy and concurrent TMZ therapy (137). In another phase 1 study, ICT-107, an autologous DC immunotherapy targeting class I peptides from TAAs expressed on gliomas and cancer stem cell populations, demonstrated promising efficacy after an intradermal administration of ICT-107 in patients with GBM (154). Furthermore, the phase II study reported an extended PFS of 2.2 months while maintaining OS compared to the unpulsed DC group. Moreover, HLA-A2-positive patients exhibited a higher immune response and greater benefit compared to HLA-A1-positive patients (137). A phase III trial is reported to be in the works, employing HLA-A2-positive, treatment-naïve GBM patients due to the clinical benefits reported by the phase 2 trial. However, the PFS improvement in the phase 2 study is very modest and the outlook is inevitably worrisome. A survival benefit for OS can also be observed in HLA-A1 patients with MGMT promoter methylation, almost doubling the PFS in the full HLA-A2 group (from 25.8 months in the control group to 47.6 months in the experimental group). These benefits underscore the need for further research in this patient subset.

Another approach involves DC vaccination pulsed with TAAs, such as Wilm’s tumour 1 peptide, which has safety and efficacy in the treatment of gliomas. A study involving patients with malignant gliomas investigated the safety and immunogenicity of WT-1 pulsed DCs vaccination therapy and reported no serious side effects, with 2 of 5 patients demonstrating tumour shrinkage (155). Recent research introduced a novel DC vaccine named CellgramDC-WT1 (CDW), which was pulsed with WT1 antigen (156). Moreover, zoledronate was selected as an inducer of DC maturation. CDW was found to induce the secretion of IL-12 and IFN-γ, which induced the differentiation of naive T-cells to active CD8+ T-cells and elicited CTL response against cancer cells with WT1 antigens (156).

#### 6.2.5 Neoantigen-based vaccines

CD8+ T-cells and CD4+ T-cells monitor peptides presented by HLA-1 and HLA-2, respectively. These peptides are also called HLA-associated peptidomes, which can provide valuable and unique information about the cell, including tumour cells. A study isolated the HLA/peptide complex from HLA-A*02+ GBM samples. They investigated 10 highly expressed glioblastoma-associated antigens in tumours and identified a multi-peptide vaccine (IMA950) (157). In a subsequent phase 1 study of IMA950 with 45 newly diagnosed GBM samples, researchers observed that 36 patients had tumour-associated peptide (TUMAP) response and 20 patients displayed multi-TUMAP response. At the primary immunogenicity endpoints, IMA950 was identified to be well tolerated, warranting further investigation (138).

Recently, personalised neoantigen-based vaccines have demonstrated excellent safety and immunogenicity in GBM. Two phase 1 clinical trials, GAPVAC-101 and the NeoVax neoantigen vaccine study, included a total of 24 patients. The median OS of GAPVAC-101 was 29 months, and both trials demonstrated high immunogenicity, with CD8+ and CD4+ T-cell responses occurring in 92% (12/13) and 80% (8/10) of patients, respectively. The GAPVAC-101 study revealed 50%−84.7% active customised vaccine-induced immunogenicity, with no occurrence of significant treatment-related AEs (139, 158). Safe and highly immunogenic personalised vaccinations targeting unmutated peptides and neoantigens hold promise for benefitting patients with GBM.

#### 6.2.6 Combination treatment

In addition to the initial successful attempts at cancer vaccines, numerous personalised cancer vaccines are currently under evaluation in clinical trials, often in combination with checkpoint blockade modulators or cytokine therapy, yielding promising results in various solid or metastatic tumours (159, 160). Notably, checkpoint inhibitor treatment can trigger tumour-reactive T-cell infiltration, which may occur spontaneously in a small number of patients with cancer who are sensitive to checkpoint blockade. Additionally, cancer vaccines activate CD8+ T-cells, increasing the expression of PD-1, CTLA-4, LAG-3 and other inhibitory receptors in T-cells (161–164).

Therefore, combination therapy with cancer vaccines and checkpoint inhibitors has promising benefits, as evidenced in multiple preclinical studies. The blockade of PD-1 and/or LAG-3 has been demonstrated to enhance the anti-tumour efficacy of vaccines in three non-glioma tumour models. In particular, in the prostate cancer model, combined blockade of PD-1 and LAG-3 with vaccination resulted in a significant anti-tumour effect. Conversely, PD-1 blockade alone, in combination with vaccination against ‘self’ tumour antigens, was less effective. Moreover, checkpoint receptor expression increased in CD8+ T-cells after vaccine-mediated activation (165).

In mice bearing intracranial gliomas, PD-1 mAb blockade combined with DC vaccination resulted in long-term survival, while neither agent alone induced a survival benefit completely dependent on CD8+ T-cells. Furthermore, DC vaccination plus PD-1 blockade resulted in TIL homing and immune memory marker upregulation. In clinical samples, DC vaccination of patients with GBM was associated with upregulated PD-1 expression *in vivo* and PD-1 blockade *in vitro*, with freshly isolated TIL significantly enhancing the cytolysis of autologous tumour cells (166).

Despite the challenges posed by the complexity of cancer immunology, ongoing research into vaccine-based cancer treatment options is encouraged by advancements in vaccine technology and our evolving understanding of cancer immunology.

### 6.3 CAR-T cells

Genetically engineered autologous T-cells expressing a chimeric antigen receptor (CAR) have revolutionised the treatment of relapsed/refractory B-cell malignancies. However, CAR-T cell therapy for solid tumours, including brain tumours, presents numerous challenges, particularly due to the BBB, which impedes T-cell localisation and effector function. However, recent clinical studies have demonstrated that CAR-T cells can successfully traffic to active GBM regions, and expand and improve treatment feasibility and safety.

CARs are bio-engineered receptors that activate T-cells to become effector cells capable of recognising and eliminating target cells expressing specific antigens (167). Target selection, coverage, specificity and target expression stability are critical considerations in CAR-T therapy efficacy (168). Current CAR-T therapeutic targets in GBM must be evaluated in light of these considerations.

#### 6.3.1 IL13Rα2 CAR-T

IL13Rα2, a high affinity IL-13 receptor monomer expressed by myeloid-derived suppressor cells and tumour-infiltrating macrophages, in over 50% of GBMs, has been associated with poor survival (168–170). Importantly, IL13Rα2 is minimally expressed in normal brain tissue (171–173), making it an attractive immunotherapy target. Recent studies have investigated CAR-T cells targeting IL13Rα2 that were delivered intracranially into the resection cavity of three patients with recurrent GBM, demonstrating good tolerance and manageable transient encephalitis. Two patients experienced transient anti-glioma responses, with one patient showing decreased IL13Rα2 expression in tumour tissue after treatment and the other exhibiting an increase in tumour necrosis volume at the administration site on MRI (174).

In another study, CAR-T cells targeting IL13Rα2 were used to treat a patient with relapsed multifocal GBM. The trial utilised two intracranial delivery methods for multiple infusions of CAR-T cells into the cavity of the excised tumour and the ventricular system. No grade 3 or higher toxicity was reported from intracranial IL13Rα2-targeted CAR-T cell infusion. After CAR-T cell therapy, all intracranial and spinal tumours retreated, and the quantities of cytokines and immune cells in the cerebrospinal fluid increased in tandem. Notably, this therapeutic effect continued for 7.5 months after CAR-T cell therapy commencement (175).

#### 6.3.2 EGFRvIII CAR-T

CAR-T targeting EGFRvIII have also been investigated for their potential in treating GBM. In a study of 10 patients with relapsed GBM, an infusion of EGFRvIII CAR-T cells resulted in detectable transient expansion of CART-EGFRvIII cells in the peripheral blood. Among them, two exhibited CART-EGFRvIII DNA levels that were three or one hundred times higher in the brain than in the peripheral blood at two weeks after infusion. However, the study also found a higher and more robust expression of inhibitory molecules in tumour samples after CART-EGFRvIII infusion, with Treg infiltration creating an opportunity for subsequent surgical treatment (176).

For improving the efficacy of EGFRvIII-targeting CAR-T without inducing off-targeting toxicity, a humanised antibody M27 was developed to specifically bind to both wild-type EGFR- and EGFRvIII-overexpressing tumour cells (177). The M27-derived CAR-T cells were found to effectively target and lyse EGFR-EGFRvIII-overexpressing tumour cells without observable toxicity on normal cells. When CD137 (4-1BB) costimulatory intracellular domain was included in the M27-28BBZ CAR-T cells, EGFR- and EGFRvIII-over-expressing GBM cells were effectively inhibited, prolonging the survival of mice (177).

Recently, a novel GCT02 CAR-T cell was designed to target a new single-chain variable fragment (scFv), demonstrating a high affinity to EGFRvIII. In a xenograft model of human GBM, GCT02 CAR-T cells rapidly eliminated tumour cells with decreased cytokines secretion (178). Further research demonstrated that GCT02 CAR-T exhibited highly specific EGFRvIII-targeting preclinical function (179). Therefore, such CAR approaches need further clinical investigations.

#### 6.3.3 HER2 CAR-T

Human epidermal growth factor receptor 2 (HER2) binds with epidermal growth factor (EGF) and transforming growth factor α (TGF-α) ligands, promoting downstream signalling pathways such as Ras/Raf/MEK/ERK1/2 and phospholipase (180). Apart from breast cancer, HER2 is also overexpressed in gastric cancer, lung cancer, oesophageal cancer and sarcomas (181, 182). Therefore, HER2 is considered an ideal CAR-targeted TAA for GBM.

Ahmed et al. reported that HER2-specific CAR-T cells from patients with GBM exhibited an anti-tumour activity against autologous primary HER2-positive GBM tumour cells and CD133-positive GBM stem cells. In an orthotopic xenogeneic SCID mouse model, these HER2-specific CAR-T cells exerted a potent anti-tumour activity (183). Similarly, in clinical research, 17 patients with advanced HER2-positive GBM tumours received one or more infusions of HER2-specific CAR-modified virus-specific T-cells (HER2-CAR VSTs). All patients had detectable HER2-CAR VSTs in peripheral blood after infusion. Although two individuals suffered from grade 2 seizures or headaches, the medication was well tolerated with no dose-limiting effects. The median OS was 11.1 months from the first T-cell infusion and 24.5 months from diagnosis. Three patients had stable disease with no evidence of progression after 24 to 29 months of follow-up (184). Thus, HER2-CAR VSTs have promising benefits in the treatment of GBM. Hence, further exploration of HER2-CAR VSTs in combination with other immunotherapy approaches might provide novel therapeutic strategies (185).

#### 6.3.4 EphA2 CAR-T

Erythropoietin-producing hepatocellular carcinoma A2 (EphA2) has emerged as an attractive target for the immunotherapy of GBM due to its overexpression in glioma while being minimally expressed i normal brain tissue. Preclinical research has shown that EphA2-specific CAR-T cells can induce regression of glioma xenografts in severe combined immunodeficiency (SCID) mice and a significant survival advantage compared to untreated mice and mice treated with nontransduced T-cells (186).

Studies have revealed that EphA2 overexpression enhances the invasiveness of GSCs *in vivo* through Akt signalling, contributing to tumour stem properties (187). Moreover, upregulation of platelet-derived growth factor (PDGF) due to high expression of EphA2, combined with PDGFA-induced EphA2 activation, correlates with worse patient prognosis and poorer therapeutic outcomes, suggesting a potential combination therapy strategy targeting both PDGFRA and EphA2 (188). In another study, CAR-T cells targeting EphA2 demonstrated anti-tumour activation linked to upregulation of CXCR-1/2 and appropriate interferon-γ (IFN-γ) production (189).

In a study of trivalent CAR-T targeting EphA2, HER2 and IL13Rα2, Bielamowicz et al. designed UCART cells that exhibited improved cytotoxicity and cytokine release and could control autologous GBM patient-derived xenografts (PDXs) models and improve survival of treated animals in low doses (190). Despite promising preclinical results, further clinical research is needed to validate their anti-tumour effects.

#### 6.3.5 Other CAR-T methods

While CAR-T therapy shows promise in glioma treatment, it has been observed that there are escape mechanisms present in single-target CAR-T therapy. For instance, when targeting HER2 in a GBM cell line, HER2-null tumour cells emerge that still express non-targeted TAAs (191). This observation highlights the need for a combined approach of targeting these TAAs to counteract this escape mechanism. Specifically, in a cohort of 20 primary GBMs, tumours targeting HER2 or IL13Rα2 alone had a near-complete eradication rate of 60%−70%, whereas tumours targeting both HER2 and IL13Rα2 had a rate exceeding 90% (191).

Based on this, Hegde M et al. designed tandem CAR-T cells (TanCAR) targeting HER2 and IL13Rα2 (192). TanCAR acts on HER2 and IL13Rα2 by inducing HER2-IL13Rα2 heterodimers and promoting superadditive T-cell activation upon simultaneous encounter of both antigens. Compared with biCAR (bi-expressers of both HER2 and IL13Rα2 CAR), HER2 CAR, IL13rα2 CAR and TanCAR exhibited higher autologous GBM cell lysis rate and cytokine production (IFN-γ and IL-2). In a GBM mouse model, TanCAR T-cells reduced antigen escape and exhibited higher anti-tumour efficacy and animal survival. Thus, TanCAR T-cells can enhance the control of glioblastoma multiforme through the synergistic effect of HER2 and IL13Rα2, highlighting their therapeutic potential (192).

Recently, a novel tandem CAR-T cell (TanCART) with dual specificity for EGFRvIII and IL-13Rα2 was reported in a preclinical study (193). TanCART exhibited more rapid and complete cytotoxicity but did not exhibit increased off-target activity against target cells compared to monospecific CAR-T cells alone. The anti-tumour activity of TanCART cells against heterogeneous glioma (U87MG) populations in an orthotopic mouse model was able to achieve long-term, complete and durable responses (193). These findings warrant further *in vivo* studies to establish a novel combination of treatment modalities that could revolutionise CAR-T cell therapy for patients with GBM.

Despite challenges in identifying an ideal target for solid tumours, exploration of new targets such as EphA2 (187, 188), GD2 (194, 195), B7-H2 (196, 197), Chlorotoxin (198) and CD317 (199) in CAR-T therapy of gliomas holds promise for developing combination treatment strategies. Additionally, CAR-T therapy should not be limited to directly destroying cancer cells. Instead, it can be leveraged to stimulate endogenous immune responses against tumours, and techniques to disrupt the tumour microenvironment may enhance efficacy.

Another consideration in CAR-T immunotherapy is the administration method. I Initial experiences with CAR-T cells in relapsed GBM suggest that both modalities of administration, either directly intracranially or via peripheral intravenous infusion, can produce targeted activity in the brain. However, there is debate regarding the optimal method, as the complete distribution of peripherally injected CAR-T cells throughout the brain, particularly in non-enhancing areas of invasive tumours, is not yet established. Another approach to the direct infusion of CAR-T cells into the CNS, such as intraluminal or intraventricular administration, appears to help reduce systemic AEs; however, relevant data are limited. 6.4 Macrophage-based immunotherapy

### 6.4 Macrophage-based Immunotherapy

Tumour-associated macrophages/microglia constitute the predominant immune cell population within the GBM microenvironment. These TAMs can be categorised into two main phenotypes: tumour-suppressing type (sTAM, M1) and tumour-promoting type (ptam, M2), based on their functional roles (200). Studies have elucidated the critical roles of pTAMs, along with glioma stem cells (GSCs), and their interplay in promoting tumour progression and therapeutic resistance in GBMs (201).

#### 6.4.1 CSF1R

CSF1R (colony-stimulating factor 1 receptor) is expressed at elevated levels in monocytes and tissue macrophages (202), regulating the differentiation and survival of the mononuclear phagocyte system and macrophages (202). PLX3397 is a small molecule, orally administered, that selectively inhibits CSF1R. In phase 2, an open-label, single-agent trial (NCT01349036), Butowski et al. demonstrated that PLX3397 was well tolerated and readily crossed the blood-tumour barrier but showed no efficacy in patients with recurrent GBM (203). Another ongoing phase 1b/2 study (NCT01790503) of PLX3397 is evaluating its potential to improve the efficacy of standard-of-care radiation therapy plus temozolomide in patients with newly diagnosed GBM.

#### 6.4.2 CD47-SIRPα

CD47, a surface immunoglobulin-like protein (204), serves various functions including regulating neutrophil migration, axon extension and T-cell co-stimulation (205). SIRPα is one of its receptors that is expressed on macrophages, which negatively regulates phagocytosis (204). Recently, Hutter et al. reported that the disruption of the SIRPα-CD47 signalling axis was effective against various brain tumours, including GBM, primarily by inducing tumour phagocytosis (206). Similarly, Hsu et al. reported that rapamycin and hydroxychloroquine (RQ) may be associated with the downregulation of the CD47-SIRPα axis, thereby reducing M2 polarisation and improving macrophage phagocytosis (207). Their data provide a rational design for GBM combined with anti-PD-1 and RQ therapy. These results provide implications for a promising therapeutic strategy in GBM by targeting the CD47- SIRPα signalling axis.

## 7 Conclusions and future perspective

Recurrence is common in HGG due to their unique biology, posing a significant hurdle in modern treatment. Recent treatment approaches have shifted towards molecular profiling of CNS tumour classifications, emphasising key genomic alterations in each classification group to guide treatment decisions and strategy development. Chemotherapy tailored to molecular characteristics offers more precise and effective treatment, with heightened chemosensitivity observed in cases with IDH mutations. There are ongoing efforts to develop advanced drug delivery systems capable of overcoming the BBB. Advances in radiation technology have enabled selective reradiation, often in combination with bevacizumab or TMZ. Antivascular targeting drugs that have shown efficacy in other solid tumours are also being explored in HGG treatment. Notably, IDH-selective inhibitors have demonstrated considerable survival benefits *in vivo*. However, checkpoint inhibitors targeting immune checkpoint expression in HGG tumour cells have yielded limited efficacy in primary and recurrent HGG compared to most solid tumours, albeit with potential for selective application. Vaccine therapy holds promise based on biological and preclinical rationale, yet its clinical translation remains challenging. Preliminary experience with CAR-T cells in relapsed GBM suggests that both direct intracranial or peripheral intravenous infusion can yield targeted activity in the brain. Future research should prioritise ideal target screening, recognising that CAR-T therapy’s role extends beyond direct tumour killing. Strategies that activate endogenous tumour immune responses and disrupt the tumour growth environment can be more effective in addressing practical challenges. Combinatorial strategies, including the combination of multiple ICIs, ICIs with vaccines and CAR-T cells, are currently at the forefront of immunotherapy to overcome the immune resistance of glioma. TTFields present a candidate for combination with immunotherapy due to their nontoxic nature, involvement in immune pathways, and activation of multiple pathways leading to apoptosis. The development of multiple treatment modalities for HGG is ongoing, necessitating further research to establish better treatment strategies and improve patient outcomes.

## Author contributions

XC: Writing – original draft, Writing – review & editing, Conceptualization, Supervision. LZ: Writing – original draft, Writing – review & editing, Supervision. YC: Writing – review & editing, Writing – original draft.

## References

[1] Ostrom QT Gittleman H Liao P Rouse C Chen Y Dowling J. CBTRUS statistical report: primary brain and central nervous system tumours diagnosed in the United States in 2007-2011. Neuro-Oncol. (2014) 16:iv1–iv63. doi: 10.1093/neuonc/nou223 25304271 PMC4193675

[2] Louis DN Perry A Wesseling P Brat DJ Cree IA Figarella-Branger D. The 2021 WHO classification of tumours of the central nervous system: A summary. Neuro-Oncology. (2021) 23:1231–51. doi: 10.1093/neuonc/noab106 PMC8328013 34185076

[3] Stupp R Weller M Belanger K Bogdahn U Ludwin SK Lacombe D. Radiotherapy plus concomitant and adjuvant temozolomide for glioblastoma. N Engl J Med. (2005) 352:987–96. doi: 10.1056/NEJMoa043330 15758009

[4] Stupp R Hegi ME Mason WP van den Bent MJ Taphoorn MJ Janzer RC. Effects of radiotherapy with concomitant and adjuvant temozolomide versus radiotherapy alone on survival in glioblastoma in a randomised phase III study: 5-year analysis of the EORTCNCIC trial. Lancet Oncol. (2009) 10:459–66. doi: 10.1016/S1470-2045(09)70025-7 19269895

[5] Friedman HS Prados MD Wen PY Mikkelsen T Schiff D Abrey LE. Bevacizumab alone and in combination with irinotecan in recurrent glioblastoma. J Clin Oncol. (2009) 27:4733–40. doi: 10.1200/JCO.2008.19.8721 19720927

[6] Stupp R Taillibert S Kanner AA Kesari S Steinberg DM Toms SA. Maintenance therapy with tumor treating fields plus temozolomide vs temozolomide alone for glioblastoma: a randomized clinical trial. JAMA. (2015) 314:2535–43. doi: 10.1001/jama.2015.16669 26670971

[7] Hegi ME Diserens AC Gorlia T Hamou MF de Tribolet N Weller M. MGMT gene silencing and benefit from temozolomide in glioblastoma. N Engl J Med. (2005) 352:997–1003. doi: 10.1056/NEJMoa043331 15758010

[8] Gilbert MR Wang M Aldape KD Stupp R Hegi ME Jaeckle KA. Dose-dense temozolomide for newly diagnosed glioblastoma: a randomized phase III clinical trial. J Clin Oncol. (2013) 31:4085–91. doi: 10.1200/JCO.2013.49.6968 PMC3816958 24101040

[9] Van den Bent MJ Baumert B Erridge SC Vogelbaum MA Nowak AK Sanson M. Interim results from the CATNON trial (EORTC study 26053-22054) of treatment with concurrent and adjuvant temozolomide for 1p/19q non-co-deleted anaplastic glioma: a phase 3, randomised, open-label intergroup study. Lancet. (2017) 390:1645–53. doi: 10.1016/S0140-6736(17)31442-3 PMC5806535 28801186

[10] Haque W Thong E Andrabi S Verma V Brian Butler E Teh BS. Prognostic and predictive impact of MGMT promoter methylation in grade 3 gliomas. J Clin Neurosci. (2021) 85:115–21. doi: 10.1016/j.jocn.2020.12.028 33581781

[11] Nordling-David MM Yaffe R Guez D Meirow H Last D Grad E. Liposomal temozolomide drug delivery using convection enhanced delivery. J Control Release. (2017) 261:138–46. doi: 10.1016/j.jconrel.2017.06.028 28666727

[12] Zhan W Wang CH. Convection enhanced delivery of chemotherapeutic drugs into brain tumour. J Control Release. (2018) 271:74–87. doi: 10.1016/j.jconrel.2017.12.020 29274437

[13] Fritzell S Sandén E Eberstål S Visse E Darabi A Siesjö P. Intratumoral temozolomide synergizes with immunotherapy in a T cell-dependent fashion. Cancer Immunol Immunother. (2013) 62:1463–74. doi: 10.1007/s00262-013-1449-z PMC11029176 23775421

[14] Kirson ED Dbalý V Tovaryš F Vymazal J Soustiel JF Itzhaki A. Alternating electric fields arrest cell proliferation in animal tumor models and human brain tumours. Proc Natl Acad Sci. (2007) 104:10152–7. doi: 10.1073/pnas.0702916104 PMC1886002 17551011

[15] Stupp R Wong ET Kanner AA Steinberg D Engelhard H Heidecke V. NovoTTF-100A versus physician’s choice chemotherapy in recurrent glioblastoma: a randomised Phase III trial of a novel treatment modality. Eur J Cancer. (2012) 48:2192–202. doi: 10.1016/j.ejca.2012.04.011 22608262

[16] Stupp R Taillibert S Kanner A Read W Steinberg D Lhermitte B. Effect of tumor-treating fields plus maintenance temozolomide vs maintenance temozolomide alone on survival in patients with glioblastoma: A randomized clinical trial. JAMA. (2017) 318:2306–16. doi: 10.1001/jama.2017.18718 PMC5820703 29260225

[17] Ram Z Kim CY Hottinger AF Idbaih A Nicholas G Zhu JJ. Efficacy and safety of tumor treating fields (TTFields) in elderly patients with newly diagnosed glioblastoma: subgroup analysis of the phase 3 EF-14 clinical trial. Front Oncol. (2021) 11:671972. doi: 10.3389/fonc.2021.671972 34692470 PMC8526342

[18] Kim CY Paek SH Nam DH Chang JH Hong YK Kim JH. Tumor treating fields plus temozolomide for newly diagnosed glioblastoma: a sub-group analysis of Korean patients in the EF-14 phase 3 trial. J Neurooncol. (2020) 146:399–406. doi: 10.1007/s11060-019-03361-2 32020470

[19] Shi W Blumenthal DT Oberheim Bush NA Kebir S Lukas RV Muragaki Y. Global post-marketing safety surveillance of Tumor Treating Fields (TTFields) in patients with high-grade glioma in clinical practice. J Neurooncol. (2020) 148:489–500. doi: 10.1007/s11060-020-03540-6 32535723 PMC7438370

[20] Onken J Staub-Bartelt F Vajkoczy P Misch M. Acceptance and compliance of TTFields treatment among high grade glioma patients. J Neurooncol. (2018) 139:177–84. doi: 10.1007/s11060-018-2858-9 29644485

[21] Diamant G Simchony Goldman H Gasri Plotnitsky L Roitman M Shiloach T Globerson-Levin A. T cells retain pivotal antitumoral functions under tumor-treating electric fields. J Immunol. (2021) 207:709–19. doi: 10.4049/jimmunol.2100100 34215656

[22] Simchony H Diamant D Ram Z Volovitz I. Evaluation of the compatibility of electric tumor treating fields with key anti-tumoral T-cell functions. Isr. Med Assoc J. (2019) 21:503. 31507132

[23] Voloshin T Kaynan N Davidi S Porat Y Shteingauz A Schneiderman RS. Tumor-treating fields (TTFields) induce immunogenic cell death resulting in enhanced antitumor efficacy when combined with anti-PD-1 therapy. Cancer Immunol Immunother. (2020) 69:1191–204. doi: 10.1007/s00262-020-02534-7 PMC7303058 32144446

[24] Bezu L Sauvat A Humeau J Leduc M Kepp O Kroemer G. eIF2α phosphorylation: a hallmark of immunogenic cell death. Oncoimmunology. (2018) 7:e1431089. doi: 10.1080/2162402X.2018.1431089 29872560 PMC5980344

[25] Fucikova J Becht E Iribarren K Goc J Remark R Damotte D. Germain, C. et al. Calreticulin Expression in Human Non-Small Cell Lung Cancers Correlates with Increased Accumulation of Antitumor Immune Cells and Favorable Prognosis. Cancer Res. (2016) 76:1746–56. doi: 10.1158/0008-5472.CAN-15-1142 26842877

[26] Fucikova J Truxova I Hensler M Becht E Kasikova L Moserova I. Calreticulin exposure by Malignant blasts correlates with robust anticancer immunity and improved clinical outcome in AML patients. Blood. (2016) 128:3113–24. doi: 10.1182/blood-2016-08-731737 PMC5201098 27802968

[27] Chen D Le SB Hutchinson TE Calinescu A-A Sebastian M Jin D. Tumor-treating fields dually activate STING and AIM2 inflammasomes to induce adjuvant immunity in glioblastoma. J Clin Invest. (2022) 132:e149258. doi: 10.1172/JCI149258 35199647 PMC9012294

[28] Kessler AF Salvador E Domro¨se D Burek M Schaeffer C Tempel Brami C. Blood brain barrier (BBB) integrity is affected by tumor treating fields (TTFields) in vitro and *in vivo* . Int J Radiat. Oncol Biol Phys. (2019) 105:162–3. doi: 10.1016/j.ijrobp.2019.06.182

[29] Scaringi C Agolli L Minniti G. Technical advances in radiation therapy for brain tumours. Anticancer Res. (2018) 38:6041–5. doi: 10.21873/anticanres.12954 30396918

[30] Kazmi F Soon YY Leong YH Koh WY Vellayappan B. Re-irradiation for recurrent glioblastoma (GBM): a systematic review and meta-analysis. J Neurooncol. (2019) 142:79–90. doi: 10.1007/s11060-018-03064-0 30523605

[31] Ciernik IF Gager Y Renner C Spieker S Arndt N Neumann K. Salvage radiation therapy for patients with relapsing glioblastoma multiforme and the role of slow fractionation. Front Oncol. (2020) 10:577443. doi: 10.3389/fonc.2020.577443 33364191 PMC7753368

[32] Kim EY Yechieli R Kim JK Mikkelsen T Kalkanis SN Rock J. Patterns of failure after radiosurgery to two different target volumes of enhancing lesions with and without FLAIR abnormalities in recurrent glioblastoma multiforme. J Neurooncol. (2014) 116:291–7. doi: 10.1007/s11060-013-1290-4 24173682

[33] Møller S Munck Af Rosenschöld P Costa J Law I Poulsen HS Engelholm SA. Toxicity and efficacy of re-irradiation of high-grade glioma in a phase I dose- and volume escalation trial. Radiother. Oncol. (2017) 125:223–7. doi: 10.1016/j.radonc.2017.09.039 29054380

[34] Chan J Jayamanne D Wheeler H Khasraw M Wong M Kastelan M. The role of large volume re-irradiation with Bevacizumab in chemorefractory high grade glioma. Clin Transl Radiat. Oncol. (2020) 22:33–9. doi: 10.1016/j.ctro.2020.03.005 PMC7075764 32195378

[35] Morris SL Zhu P Rao M Martir M Zhu JJ Hsu S. Gamma knife stereotactic radiosurgery in combination with bevacizumab for recurrent glioblastoma. World Neurosurg. (2019) 127:e523–e33. doi: 10.1016/j.wneu.2019.03.193 30954746

[36] Tsien C Pugh S Dicker AP Raizer JJ Matuszak MM Lallana E. Randomized phase II trial of re-irradiation and concurrent bevacizumab versus bevacizumab alone as treatment for recurrent glioblastoma (NRG Oncology/RTOG 1205): initial outcomes and RT plan quality report. Int J Radiat. Oncol Biol Phys. (2019) 105:S78. doi: 10.1016/j.ijrobp.2019.06.539

[37] Navarria P Minniti G Clerici E Tomatis S Pinzi V Ciammella P. Re-irradiation for recurrent glioma: outcome evaluation, toxicity and prognostic factors assessment. a multicenter study of the Radiation Oncology Italian Association (AIRO). J Neurooncol. (2019) 142:59–67. doi: 10.1007/s11060-018-03059-x 30515706

[38] Le Rhun E Preusser M Roth P Reardon DA van den Bent M Wen P. Molecular targeted therapy of glioblastoma. Cancer Treat Rev. (2019) 80:101896. doi: 10.1016/j.ctrv.2019.101896 31541850

[39] Kreisl TN Kim L Moore K Duic P Royce C Stroud I. Phase II trial of single-agent bevacizumab followed by bevacizumab plus irinotecan at tumor progression in recurrent glioblastoma. J Clin Oncol. (2009) 27:740–5. doi: 10.1200/JCO.2008.16.3055 PMC2645088 19114704

[40] Ameratunga M Pavlakis N Wheeler H Grant R Simes J Khasraw M. Anti-angiogenic therapy for high-grade glioma. Cochrane Database Syst Rev. (2018) 11:CD008218. doi: 10.1002/14651858.CD008218.pub4 30480778 PMC6516839

[41] Diaz RJ Ali S Qadir MG de la Fuente MI Ivan ME Komotar RJ. The role of bevacizumab in the treatment of glioblastoma. J Neurooncol. (2017) 133:455–67. doi: 10.1007/s11060-017-2477-x 28527008

[42] Peng H Zhang Q Li J Zhang N Hua Y Xu L. Apatinib inhibits VEGF signaling and promotes apoptosis in intrahepatic cholangiocarcinoma. Oncotarget. (2016) 7:17220–9. doi: 10.18632/oncotarget.v7i13 PMC4941382 26967384

[43] Xia L Gong M Zou Y Wang Z Wu B Zhang S. Apatinib induces ferroptosis of glioma cells through modulation of the VEGFR2/nrf2 pathway. Oxid Med Cell Longev. (2022) 2022:9925919. doi: 10.1155/2022/9925919 35602105 PMC9117021

[44] Wang C Jiang M Hou H Lin Q Yan Z Zhang X. Apatinib suppresses cell growth and metastasis and promotes antitumor activity of temozolomide in glioma. Oncol Lett. (2018) 16:5607–14. doi: 10.3892/ol PMC6176256 30344715

[45] Yao H Liu J Zhang C Shao Y Li X Feng M. Clinical study of apatinib plus temozolomide for the treatment of recurrent high-grade gliomas. J Clin Neurosci. (2021) 90:82–8. doi: 10.1016/j.jocn.2021.05.032 34275586

[46] Molinaro AM Taylor JW Wiencke JK Wrensch MR. Genetic and molecular epidemiology of adult diffuse glioma. Nat Rev Neurol. (2019) 15:405–17. doi: 10.1038/s41582-019-0220-2 PMC7286557 31227792

[47] Reitman ZJ Yan H. Isocitrate dehydrogenase 1 and 2 mutations in cancer: alterations at a crossroads of cellular metabolism. J Natl Cancer Inst. (2010) 102:932–41. doi: 10.1093/jnci/djq187 PMC2897878 20513808

[48] Waitkus MS Diplas BH Yan H. Isocitrate dehydrogenase mutations in gliomas. Neuro-Oncol. (2016) 18:16–26. doi: 10.1093/neuonc/nov136 26188014 PMC4677412

[49] Ye D Guan KL Xiong Y. Metabolism, activity, and targeting of D- and L-2-hydroxyglutarates. Trends Cancer. (2018) 4:151–65. doi: 10.1016/j.trecan.2017.12.005 PMC5884165 29458964

[50] Natsume A Arakawa Y Narita Y Sugiyama K Hata N Muragaki Y. The first-in-human phase i study of a brain-penetrant mutant IDH1 inhibitor DS-1001 in patients with recurrent or progressive IDH1-mutant gliomas. Neuro Oncol. (2023) 25:326–36. doi: 10.1093/neuonc/noac155 PMC9925696 35722822

[51] Watts JM Baer MR Yang J Prebet T Lee S Schiller GJ. Olutasidenib alone or with azacitidine in IDH1-mutated acute myeloid leukaemia and myelodysplastic syndrome: phase 1 results of a phase 1/2 trial. Lancet Haematol. (2023) 10:e46–58. doi: 10.1016/S2352-3026(22)00292-7 PMC12250719 36370742

[52] Mellinghoff IK Penas-Prado M Peters KB Burris HA Maher EA Janku F. Vorasidenib, a dual inhibitor of mutant IDH1/2, in recurrent or progressive glioma; results of a first-in-Human phase i trial. Clin Cancer Res. (2021) 27:4491–9. doi: 10.1158/1078-0432.CCR-21-0611 PMC8364866 34078652

[53] Pusch S Krausert S Fischer V Balss J Ott M Schrimpf D. Pan-mutant IDH1 inhibitor BAY 1436032 for effective treatment of IDH1 mutant astrocytoma in *vivo* . Acta Neuropathol. (2017) 133:629–44. doi: 10.1007/s00401-017-1677-y 28124097

[54] Kopinja J Sevilla RS Levitan D Dai D Vanko A Spooner E. A brain penetrant mutant IDH1 inhibitor provides in vivo survival benefit. Sci Rep. (2017) 7:13853. doi: 10.1038/s41598-017-14065-w 29062039 PMC5653818

[55] Oh S Yeom J Cho HJ Kim JH Yoon SJ Kim H. Integrated pharmaco-proteogenomics defines two subgroups in isocitrate dehydrogenase wild-type glioblastoma with prognostic and therapeutic opportunities. Nat Commun. (2020) 11:3288. doi: 10.1038/s41467-020-17139-y 32620753 PMC7335111

[56] Goldmann J Kwidzinski E Brandt C Mahlo J Richter D Bechmann I. T cells traffic from brain to cervical lymph nodes via the cribroid plate and the nasal mucosa. J Leukoc Biol. (2006) 80:797–801. doi: 10.1189/jlb.0306176 16885505

[57] Davies DC. Blood–brain barrier breakdown in septic encephalopathy and brain tumours. J Anat. (20) 200:639–46. doi: 10.1046/j.1469-7580.2002.00065.x PMC1570752 12162731

[58] Kamran N Alghamri MS Nunez FJ Shah D Asad AS Candolfi M. Current state and future prospects of immunotherapy for glioma. Immunotherapy. (2018) 10:317–39. doi: 10.2217/imt-2017-0122 PMC5810852 29421984

[59] Lécuyer M-A Kebir H Prat A. Glial influences on BBB functions and molecular players in immune cell trafficking. Biochim Biophys Acta. (2016) 1862:472–82. doi: 10.1016/j.bbadis.2015.10.004 26454208

[60] Louveau A Smirnov I Keyes TJ Eccles JD Rouhani SJ Peske JD. Structural and functional features of central nervous system lymphatic vessels. Nature. (2015) 523:337–41. doi: 10.1038/nature14432 PMC4506234 26030524

[61] Marchetti L Engelhardt B. Immune cell trafficking across the blood-brain barrier in the absence and presence of neuroinflammation. Vasc Biol. (2020) 2:H1–18. doi: 10.1530/VB-19-0033 32923970 PMC7439848

[62] Nirschl CJ Drake CG. Molecular pathways: coexpression of immune checkpoint molecules: signaling pathways and implications for cancer immunotherapy. Clin Cancer Res. (2013) 19:4917–24. doi: 10.1158/1078-0432.CCR-12-1972 PMC4005613 23868869

[63] Pardoll DM. The blockade of immune checkpoints in cancer immunotherapy. Nat Rev Cancer. (2012) 12:252–64. doi: 10.1038/nrc3239 PMC4856023 22437870

[64] Carlino MS Larkin J Long GV. Immune checkpoint inhibitors in melanoma. Lancet. (2021) 398:1002–14. doi: 10.1016/S0140-6736(21)01206-X 34509219

[65] Gong J Chehrazi-Raffle A Reddi S Salgia R. Development of PD-1 and PD-L1 inhibitors as a form of cancer immunotherapy: a comprehensive review of registration trials and future considerations. J Immunother Cancer. (2018) 6:8. doi: 10.1186/s40425-018-0316-z 29357948 PMC5778665

[66] Zeng J Zhang XK Chen HD Zhong ZH Wu QL Lin SX. Expression of programmed cell death-ligand 1 and its correlation with clinical outcomes in gliomas. Oncotarget. (2016) 7:8944–55. doi: 10.18632/oncotarget.v7i8 PMC4891016 26771840

[67] Han J Hong Y Lee YS. PD-L1 expression and combined status of PD-L1/PD-1-positive tumor infiltrating mononuclear cell density predict prognosis in glioblastoma patients. J Pathol Transl Med. (2017) 51:40–8. doi: 10.4132/jptm.2016.08.31 PMC5267537 27989100

[68] Wang Z Zhang C Liu X Wang Z Sun L Li G. Molecular and clinical characterization of PD-L1 expression at transcriptional level *via* 976 samples of brain glioma. Oncoimmunology. (2016) 5:e1196310. doi: 10.1080/2162402X.2016.1196310 27999734 PMC5139638

[69] Lee KS Lee K Yun S Moon S Park Y Han JH. Prognostic relevance of programmed cell death ligand 1 expression in glioblastoma. J Neurooncol. (2018) 136:453–61. doi: 10.1007/s11060-017-2675-6 29147863

[70] Berghoff AS Kiesel B Widhalm G Wilhelm D Rajky O Kurscheid S. Correlation of immune phenotype with IDH mutation in diffuse glioma. Neuro-Oncol. (2017) 19:1460–8. doi: 10.1093/neuonc/nox054 PMC5737620 28531337

[71] Liu C Zhang Z Ping Y Qin G Zhang K Maimela NR. Comprehensive analysis of PD-1 gene expression, immune characteristics and prognostic significance in 1396 glioma patients. Cancer Manage Res. (2020) 12:4399–410. doi: 10.2147/CMAR.S238174 PMC7294103 32606935

[72] Berghoff AS Kiesel B Widhalm G Rajky O Ricken G Wöhrer A. Programmed death ligand 1 expression and tumor-infiltrating lymphocytes in glioblastoma. Neuro-Oncol. (2015) 17:1064–75. doi: 10.1093/neuonc/nou307 PMC4490866 25355681

[73] Nduom EK Wei J Yaghi NK Huang N Kong LY Gabrusiewicz K. PD-L1 expression and prognostic impact in glioblastoma. Neuro-Oncol. (2016) 18:195–205. doi: 10.1093/neuonc/nov172 26323609 PMC4724183

[74] Ding XC Wang LL Zhang XD Xu JL Li PF Liang H. The relationship between expression of PD-L1 and HIF-1α in glioma cells under hypoxia. J Hematol Oncol. (2021) 14:92. doi: 10.1186/s13045-021-01102-5 34118979 PMC8199387

[75] Nayak L Molinaro AM Peters K Clarke JL Jordan JT de Groot J. Randomized Phase II and Biomarker Study of Pembrolizumab plus Bevacizumab versus Pembrolizumab Alone for Patients with Recurrent Glioblastoma. Clin Cancer Res. (2021) 27:1048–57. doi: 10.1158/1078-0432.CCR-20-2500 PMC8284901 33199490

[76] Reiss SN Yerram P Modelevsky L Grommes C. Retrospective review of safety and efficacy of programmed cell death-1 inhibitors in refractory high grade gliomas. J Immunother. Cancer. (2017) 5:99. doi: 10.1186/s40425-017-0302-x 29254497 PMC5735528

[77] Kurz SC Cabrera LP Hastie D Huang R Unadkat P Rinne M. PD-1 inhibition has only limited clinical benefit in patients with recurrent high-grade glioma. Neurology. (2018) 91:e1355–9. doi: 10.1212/WNL.0000000000006283 30171077

[78] Reardon DA Brandes AA Omuro A Mulholland P Lim M Wick A. Effect of nivolumab vs bevacizumab in patients with recurrent glioblastoma: the checkMate 143 phase 3 randomized clinical trial. JAMA Oncol. (2020) 6:1003. doi: 10.1001/jamaoncol.2020.1024 32437507 PMC7243167

[79] Cloughesy TF Mochizuki AY Orpilla JR Hugo W Lee AH Davidson TB. Neoadjuvant anti-PD-1 immunotherapy promotes a survival benefit with intratumoral and systemic immune responses in recurrent glioblastoma. Nat Med. (2019) 25:477–86. doi: 10.1038/s41591-018-0337-7 PMC6408961 30742122

[80] Schalper KA Rodriguez-Ruiz ME Diez-Valle R López-Janeiro A Porciuncula A Idoate MA. Neoadjuvant nivolumab modifies the tumor immune microenvironment in resectable glioblastoma. Nat Med. (2019) 25:470–6. doi: 10.1038/s41591-018-0339-5 30742120

[81] Lee AH Sun L Mochizuki AY Reynoso JG Orpilla J Chow F. Neoadjuvant PD-1 blockade induces T cell and cDC1 activation but fails to overcome the immunosuppressive tumor associated macrophages in recurrent glioblastoma. Nat Commun. (2021) 12:6938. doi: 10.1038/s41467-021-26940-2 34836966 PMC8626557

[82] Mirzaei R Sarkar S Yong VW. T cell exhaustion in glioblastoma: intricacies of immune checkpoints. Trends Immunol. (2017) 38:104–15. doi: 10.1016/j.it.2016.11.005 27964820

[83] Gustafson MP Lin Y New KC Bulur PA O'Neill BP Gastineau DA. Systemic immune suppression in glioblastoma: the interplay between CD14+HLA-DRlo/ neg monocytes, tumor factors, and dexamethasone. Neuro-Oncol. (2010) 12:631–44. doi: 10.1093/neuonc/noq001 PMC2940665 20179016

[84] Banks WA. Characteristics of compounds that cross the blood-brain barrier. BMC Neurol. (2009) 9:S3. doi: 10.1186/1471-2377-9-S1-S3 19534732 PMC2697631

[85] Filley AC Henriquez M Dey M. Recurrent glioma clinical trial, CheckMate-143: the game is not over yet. Oncotarget. (2017) 8:91779–94. doi: 10.18632/oncotarget.v8i53 PMC5710964 29207684

[86] Chen L. Co-inhibitory molecules of the B7–CD28 family in the control of T-cell immunity. Nat Rev Immunol. (2004) 4:336–47. doi: 10.1038/nri1349 15122199

[87] Krummel MF Allison JP. CD28 and CTLA-4 have opposing effects on the response of T cells to stimulation. J Exp Med. (1995) 182:459–65. doi: 10.1084/jem.182.2.459 PMC2192127 7543139

[88] Liu F Huang J Liu X Cheng Q Luo C Liu Z. CTLA-4 correlates with immune and clinical characteristics of glioma. Cancer Cell Int. (2020) 20:7. doi: 10.1186/s12935-019-1085-6 31911758 PMC6945521

[89] Mahmoud AB Ajina R Aref S Darwish M Alsayb M Taher M. Advances in immunotherapy for glioblastoma multiforme. Front Immunol. (2022) 13:944452. doi: 10.3389/fimmu.2022.944452 36311781 PMC9597698

[90] Brown NF Ng SM Brooks C Coutts T Holmes J Roberts C. A phase II open label, randomised study of ipilimumab with temozolomide versus temozolomide alone after surgery and chemoradiotherapy in patients with recently diagnosed glioblastoma: the Ipi-Glio trial protocol. BMC Cancer. (2020) 20:198. doi: 10.1186/s12885-020-6624-y 32164579 PMC7068928

[91] Mulholland PJ Brown NF McBain C Brazil L Peoples S Jefferies S. A randomised phase II multicentre study of ipilimumab with temozolomide vs temozolomide alone after surgery and chemoradiotherapy in patients with recently diagnosed glioblastoma: Ipi-Glio. JCO. (2023) 41:LBA2023–LBA2023. doi: 10.1200/JCO.2023.41.17_suppl.LBA2023

[92] Duerinck J Schwarze JK Awada G Tijtgat J Vaeyens F Bertels C. Intracerebral administration of CTLA-4 and PD-1 immune checkpoint blocking monoclonal antibodies in patients with recurrent glioblastoma: a phase I clinical trial. J Immunother Cancer. (2021) 9:e002296. doi: 10.1136/jitc-2020-002296 34168003 PMC8231061

[93] Anderson AC. Tim-3, a negative regulator of anti-tumour immunity. Curr Opin Immunol. (2012) 24:213–6. doi: 10.1016/j.coi.2011.12.005 22226204

[94] Liu Z Han H He X Li S Wu C Yu C. Expression of the galectin-9-Tim-3 pathway in glioma tissues is associated with the clinical manifestations of glioma. Oncol Let. (2016) 11:1829–34. doi: 10.3892/ol.2016.4142 PMC4774531 26998085

[95] Kim JE Patel MA Mangraviti A Kim ES Theodros D Velarde E. Combination therapy with anti-PD-1, anti-TIM-3, and focal radiation results in regression of murine gliomas. Clin Cancer Res. (2017) 23:124–36. doi: 10.1158/1078-0432.CCR-15-1535 PMC5735836 27358487

[96] Desai J Meniawy T Beagle B Li Z Mu S Wu J. Bgb-A425, an investigational anti-TIM-3 monoclonal antibody, in combination with tislelizumab, an anti-PD-1 monoclonal antibody, in patients with advanced solid tumours: A phase I/II trial in progress. JCO. (2020) 38:TPS3146–TPS3146. doi: 10.1200/JCO.2020.38.15_suppl.TPS3146

[97] Andrews LP Marciscano AE Drake CG Vignali DAA. LAG 3 ( CD 223) as a cancer immunotherapy target. Immunol Rev. (2017) 276:80–96. doi: 10.1111/imr.12519 28258692 PMC5338468

[98] Kisielow M Kisielow J Capoferri-Sollami G Karjalainen K. Expression of lymphocyte activation gene 3 (LAG-3) on B cells is induced by T cells. Eur J Immunol. (2005) 35:2081–8. doi: 10.1002/(ISSN)1521-4141 15971272

[99] He Y Rivard CJ Rozeboom L Yu H Ellison K Kowalewski A. Lymphocyte-activation gene-3, an important immune checkpoint in cancer. Cancer Science. (2016) 107:1193–7. doi: 10.1111/cas.12986 PMC5021038 27297395

[100] Harris-Bookman S Mathios D Martin AM Xia Y Kim E Xu H. Expression of LAG-3 and efficacy of combination treatment with anti-LAG-3 and anti-PD-1 monoclonal antibodies in glioblastoma. Intl J Cancer. (2018) 143:3201–8. doi: 10.1002/ijc.31661 PMC7105259 30248181

[101] Mair MJ Kiesel B Feldmann K Widhalm G Dieckmann K Wöhrer A. LAG-3 expression in the inflammatory microenvironment of glioma. J Neurooncol. (2021) 152:533–9. doi: 10.1007/s11060-021-03721-x PMC8084780 33651248

[102] Lynes J Jackson S Sanchez V Dominah G Wang X Kuek A. Cytokine microdialysis for real-time immune monitoring in glioblastoma patients undergoing checkpoint blockade. Neurosurg. (2019) 84:945–53. doi: 10.1093/neuros/nyy392 PMC6500883 30189044

[103] Deaglio S Dwyer KM Gao W Friedman D Usheva A Erat A. Adenosine generation catalyzed by CD39 and CD73 expressed on regulatory T cells mediates immune suppression. J Exp Med. (2007) 204:1257–65. doi: 10.1084/jem.20062512 PMC2118603 17502665

[104] Kaur T Weadick B Mace TA Desai K Odom H Govindarajan R. Nucleoside transporters and immunosuppressive adenosine signalling in the tumour microenvironment: Potential therapeutic opportunities. Pharmacol Ther. (2022) 240:108300. doi: 10.1016/j.pharmthera.2022.108300 36283452 PMC10290419

[105] Xu S Shao Q-Q Sun J-T Yang N Xie Q Wang D-H. Synergy between the ectoenzymes CD39 and CD73 contributes to adenosinergic immunosuppression in human Malignant gliomas. Neuro Oncol. (2013) 15:1160–72. doi: 10.1093/neuonc/not067 PMC3748917 23737488

[106] Markman B Hsieh AH-C Coward J Carlino MS Frentzas S Jin X. A phase I study of AK119, an anti-CD73 monoclonal antibody, in combination with AK104, an anti-PD-1/CTLA-4 bispecific antibody, in patients with advanced or metastatic solid tumours. JCO. (2021) 39:TPS2675–TPS2675. doi: 10.1200/JCO.2021.39.15_suppl.TPS2675

[107] Melero I Murillo O Dubrot J Hervás-Stubbs S Perez-Gracia JL. Multi-layered action mechanisms of CD137 (4-1BB)-targeted immunotherapies. Trends Pharmacol Sci. (2008) 29:383–90. doi: 10.1016/j.tips.2008.05.005 18599129

[108] Vinay DS Kwon BS. Immunotherapy of cancer with 4-1BB. Mol Cancer Ther. (2012) 11:1062–70. doi: 10.1158/1535-7163.MCT-11-0677 22532596

[109] Kühnöl C Herbarth M Föll J Staege MS Kramm C. CD137 stimulation and p38 MAPK inhibition improve reactivity in an in vitro model of glioblastoma immunotherapy. Cancer Immunol Immunother. (2013) 62:1797–809. doi: 10.1007/s00262-013-1484-9 PMC11028552 24129764

[110] Puigdelloses M Garcia-Moure M Labiano S Laspidea V Gonzalez-Huarriz M Zalacain M. CD137 and PD-L1 targeting with immunovirotherapy induces a potent and durable anti-tumour immune response in glioblastoma models. J Immunother Cancer. (2021) 9:e002644. doi: 10.1136/jitc-2021-002644 34281988 PMC8291319

[111] Yu X Harden K Gonzalez LC Francesco M Chiang E Irving B. The surface protein TIGIT suppresses T cell activation by promoting the generation of mature immunoregulatory dendritic cells. Nat Immunol. (2009) 10:48–57. doi: 10.1038/ni.1674 19011627

[112] Johnston RJ Comps-Agrar L Hackney J Yu X Huseni M Yang Y. The immunoreceptor TIGIT regulates anti-tumour and antiviral CD8(+) T cell effector function. Cancer Cell. (2014) 26:923–37. doi: 10.1016/j.ccell.2014.10.018 25465800

[113] Kurtulus S Sakuishi K Ngiow S-F Joller N Tan DJ Teng MWL. TIGIT predominantly regulates the immune response. via Regul T Cells J Clin Invest. (2015) 125:4053–62. doi: 10.1172/JCI81187 PMC4639980 26413872

[114] Lucca LE Lerner BA Park C DeBartolo D Harnett B Kumar VP. Differential expression of the T-cell inhibitor TIGIT in glioblastoma and MS. Neurol Neuroimmunol Neuroinflamm. (2020) 7:e712. doi: 10.1212/NXI.0000000000000712 32269065 PMC7188477

[115] Hung AL Maxwell R Theodros D Belcaid Z Mathios D Luksik AS. TIGIT and PD-1 dual checkpoint blockade enhances anti-tumour immunity and survival in GBM. Oncoimmunology. (2018) 7:e1466769. doi: 10.1080/2162402X.2018.1466769 30221069 PMC6136875

[116] Raphael I Kumar R McCarl LH Shoger K Wang L Sandlesh P. TIGIT and PD-1 immune checkpoint pathways are associated with patient outcome and anti-tumour immunity in glioblastoma. Front Immunol. (2021) 12:637146. doi: 10.3389/fimmu.2021.637146 34025646 PMC8137816

[117] Wang C Feng H Cheng X Liu K Cai D Zhao R. Potential therapeutic targets of B7 family in colorectal cancer. Front Immunol. (2020) 11:681. doi: 10.3389/fimmu.2020.00681 32477326 PMC7232583

[118] Qi Y Liu B Sun Q Xiong X Chen Q. Immune checkpoint targeted therapy in glioma: status and hopes. Front Immunol. (2020) 11:578877. doi: 10.3389/fimmu.2020.578877 33329549 PMC7729019

[119] Chapoval AI Ni J Lau JS Wilcox RA Flies DB Liu D. B7-H3: a costimulatory molecule for T cell activation and IFN-gamma production. Nat Immunol. (2001) 2:269–74. doi: 10.1038/85339 11224528

[120] Prasad DVR Nguyen T Li Z Yang Y Duong J Wang Y. Murine B7-H3 is a negative regulator of T cells. J Immunol. (2004) 173:2500–6. doi: 10.4049/jimmunol.173.4.2500 15294965

[121] Wang Z Wang Z Zhang C Liu X Li G Liu S. Genetic and clinical characterization of B7-H3 (CD276) expression and epigenetic regulation in diffuse brain glioma. Cancer Sci. (2018) 109:2697–705. doi: 10.1111/cas.13744 PMC6125452 30027617

[122] Dai L Guo X Xing Z Tao Y Liang W Shi Z. Multi-omics analyses of CD276 in pan-cancer reveals its clinical prognostic value in glioblastoma and other major cancer types. BMC Cancer. (2023) 23:102. doi: 10.1186/s12885-023-10575-1 36717836 PMC9885708

[123] Wang J-Y Wang W-P. B7-H4, a promising target for immunotherapy. Cell Immunol. (2020) 347:104008. doi: 10.1016/j.cellimm.2019.104008 31733822

[124] Yao Y Ye H Qi Z Mo L Yue Q Baral A. B7-H4(B7x)-mediated cross-talk between glioma-initiating cells and macrophages *via* the IL6/JAK/STAT3 pathway lead to poor prognosis in glioma patients. Clin Cancer Res. (2016) 22:2778–90. doi: 10.1158/1078-0432.CCR-15-0858 PMC4891287 27001312

[125] Chen D Li G Ji C Lu Q Qi Y Tang C. Enhanced B7-H4 expression in gliomas with low PD-L1 expression identifies super-cold tumours. J Immunother Cancer. (2020) 8:e000154. doi: 10.1136/jitc-2019-000154 32457124 PMC7253052

[126] ElTanbouly MA Croteau W Noelle RJ Lines JL. VISTA: a novel immunotherapy target for normalizing innate and adaptive immunity. Semin Immunol. (2019) 42:101308. doi: 10.1016/j.smim.2019.101308 31604531 PMC7233310

[127] Ghouzlani A Lakhdar A Rafii S Karkouri M Badou A. The immune checkpoint VISTA exhibits high expression levels in human gliomas and associates with a poor prognosis. Sci Rep. (2021) 11:21504. doi: 10.1038/s41598-021-00835-0 34728682 PMC8563991

[128] Wang Y Li M Wang G Wu H. Role of B7 family members in glioma: Promising new targets for tumour immunotherapy. Front Oncol. (2023) 12:1091383. doi: 10.3389/fonc.2022.1091383 36741734 PMC9890054

[129] Jiang T Wu W Zhang H Zhang X Zhang D Wang Q. High expression of B7-H6 in human glioma tissues promotes tumour progression. Oncotarget. (2017) 8:37435–47. doi: 10.18632/oncotarget.v8i23 PMC5514920 28415577

[130] Che F Xie X Wang L Su Q Jia F Ye Y. B7-H6 expression is induced by lipopolysaccharide and facilitates cancer invasion and metastasis in human gliomas. Int Immunopharmacol. (2018) 59:318–27. doi: 10.1016/j.intimp.2018.03.020 29679856

[131] Liu W Tang H Li L Wang X Yu Z Li J. Peptide-based therapeutic cancer vaccine: Current trends in clinical application. Cell Prolif. (2021) 54:e13025. doi: 10.1111/cpr.13025 33754407 PMC8088465

[132] Weller M Butowski N Tran DD Recht LD Lim M Hirte H. Rindopepimut with temozolomide for patients with newly diagnosed, EGFRvIII-expressing glioblastoma (ACT IV): a randomised, double-blind, international phase 3 trial. Lancet Oncol. (2017) 18:1373–85. doi: 10.1016/S1470-2045(17)30517-X 28844499

[133] Reardon DA Desjardins A Vredenburgh JJ O'Rourke DM Tran DD Fink KL. Rindopepimut with bevacizumab for patients with relapsed EGFRvIII-expressing glioblastoma (ReACT): results of a double-blind randomized phase II trial. Clin Cancer Res. (2020) 26:1586–94. doi: 10.1158/1078-0432.CCR-18-1140 32034072

[134] Platten M Bunse L Wick A Bunse T Le Cornet L Harting I. A vaccine targeting mutant IDH1 in newly diagnosed glioma. Nature. (2021) 592:463–8. doi: 10.1038/s41586-021-03363-z PMC8046668 33762734

[135] Izumoto S Tsuboi A Oka Y Suzuki T Hashiba T Kagawa N. Phase II clinical trial of Wilms tumor 1 peptide vaccination for patients with recurrent glioblastoma multiforme. J neurosurg. (2008) 108:963–71. doi: 10.3171/JNS/2008/108/5/0963 18447714

[136] Tsuboi A Hashimoto N Fujiki F Morimoto S Kagawa N Nakajima H. A phase I clinical study of a cocktail vaccine of Wilms' tumor 1 (WT1) HLA class I and II peptides for recurrent Malignant glioma. Cancer immunol immunother CII. (2019) 68:331–40. doi: 10.1007/s00262-018-2274-1 PMC6394509 30430205

[137] Wen PY Reardon DA Armstrong TS Phuphanich S Aiken RD Landolfi JC. A randomized double-blind placebo-controlled phase II trial of dendritic cell vaccine ICT-107 in newly diagnosed patients with glioblastoma. Clin Cancer Res. (2019) 25:5799–807. doi: 10.1158/1078-0432.CCR-19-0261 PMC8132111 31320597

[138] Rampling R Peoples S Mulholland PJ James A Al-Salihi O Twelves CJ. A cancer research UK first time in human phase I trial of IMA950 (Novel multipeptide therapeutic vaccine) in patients with newly diagnosed glioblastoma. Clin Cancer Res an Off J Am Assoc Cancer Res. (2016) 22:4776–85. doi: 10.1158/1078-0432.CCR-16-0506 PMC5026298 27225692

[139] Hilf N Kuttruff-Coqui S Frenzel K Bukur V Stevanović S Gouttefangeas C. Actively personalized vaccination trial for newly diagnosed glioblastoma. Nature. (2019) 565:240–5. doi: 10.1038/s41586-018-0810-y 30568303

[140] Ekstrand AJ Sugawa N James CD Collins VP. Amplified and rearranged epidermal growth factor receptor genes in human glioblastomas reveal deletions of sequences encoding portions of the N- and/or C-terminal tails. Proc Natl Acad Sci. (1992) 89:4309–13. doi: 10.1073/pnas.89.10.4309 PMC49071 1584765

[141] Sugawa N Ekstrand AJ James CD Collins VP. Identical splicing of aberrant epidermal growth factor receptor transcripts from amplified rearranged genes in human glioblastomas. Proc Natl Acad Sci. (1990) 87:8602–6. doi: 10.1073/pnas.87.21.8602 PMC55005 2236070

[142] Bagley SJ Desai AS Linette GP June CH O'Rourke DM. CAR T-cell therapy for glioblastoma: recent clinical advances and future challenges. Neuro-Oncol. (2018) 20:1429–38. doi: 10.1093/neuonc/noy032 PMC6176794 29509936

[143] Heimberger AB Crotty LE Archer GE Hess KR Wikstrand CJ Friedman AH. Epidermal growth factor receptor VIII peptide vaccination is efficacious against established intracerebral tumors. Clin Cancer Res. (2003) 9:4247–54. 14519652

[144] Fidanza M Gupta P Sayana A Shanker V Pahlke SM Vu B. Enhancing proteasomal processing improves survival for a peptide vaccine used to treat glioblastoma. Sci Transl Med. (2021) 13:eaax4100. doi: 10.1126/scitranslmed.aax4100 34135109 PMC13268099

[145] Schumacher T Bunse L Pusch S Sahm F Wiestler B Quandt J. A vaccine targeting mutant IDH1 induces antitumour immunity. Nature. (2014) 512:324–7. doi: 10.1038/nature13387 25043048

[146] Bunse L Schumacher T Sahm F Pusch S Oezen I Rauschenbach K. Proximity ligation assay evaluates IDH1R132H presentation in gliomas. J Clin Invest. (2015) 125:593–606. doi: 10.1172/JCI77780 25555220 PMC4319432

[147] Melief CJ. Mutation-specific T cells for immunotherapy of gliomas. N Engl J Med. (2015) 372:1956–8. doi: 10.1056/NEJMcibr1501818 25970054

[148] Pellegatta S Valletta L Corbetta C Patanè M Zucca I Riccardi Sirtori F. Effective immuno-targeting of the IDH1 mutation R132H in a murine model of intracranial glioma. Acta Neuropathol. Commun. (2015) 3:4. doi: 10.1186/s40478-014-0180-0 25849072 PMC4359524

[149] Bunse L Pusch S Bunse T Sahm F Sanghvi K Friedrich M. Suppression of antitumor T cell immunity by the oncometabolite (R)- 2-hydroxyglutarate. Nat Med. (2018) 24:1192–203. doi: 10.1038/s41591-018-0095-6 29988124

[150] Cordner R Jhun M Panwar A Wang H Gull N Murali R. Glioma immunotherapy enhancement and CD8-specific sialic acid cleavage by isocitrate dehydrogenase (IDH)-1. Oncogene. (2023) 42:2088–98. doi: 10.1038/s41388-023-02713-7 PMC10275753 37161052

[151] Hashiba T Izumoto S Kagawa N Suzuki T Hashimoto N Maruno M. Expression of WT1 protein and correlation with cellular proliferation in glial tumors. Neurologia medico-chirurgica. (2007) 47:165–70. doi: 10.2176/nmc.47.165 17457020

[152] Bregy A Wong TM Shah AH Goldberg JM Komotar RJ. Active immunotherapy using dendritic cells in the treatment of glioblastoma multiforme. Cancer Treat Rev. (2013) 39:891–907. doi: 10.1016/j.ctrv.2013.05.007 23790634

[153] Cao JX Zhang XY Liu JL Li D Li JL Liu YS. Clinical efficacy of tumor antigen-pulsed DC treatment for high-grade glioma patients: evidence from a meta-analysis. PloS One. (2014) 9:e107173. doi: 10.1371/journal.pone.0107173 25215607 PMC4162602

[154] Phuphanich S Wheeler CJ Rudnick JD Mazer M Wang H Nuno MA. Phase I trial of a multi-epitope-pulsed dendritic cell vaccine for patients with newly diagnosed glioblastoma. Cancer Immunol Immunother. (2013) 62:125–35. doi: 10.1007/s00262-012-1319-0 PMC3541928 22847020

[155] Sakai K Shimodaira S Maejima S Udagawa N Sano K Higuchi Y. Dendritic cell-based immunotherapy targeting Wilms' tumor 1 in patients with recurrent Malignant glioma. J neurosurg. (2015) 123:989–97. doi: 10.3171/2015.1.JNS141554 26252465

[156] Cho SY Jeong SM Jeon YJ Yang SJ Hwang JE Yoo BM. WT1 pulsed human CD141+ Dendritic cell vaccine has high potential in solid tumor-targeted immunotherapy. Int J Mol Sci. (2023) 24:1501. doi: 10.3390/ijms24021501 36675017 PMC9864659

[157] Dutoit V Herold-Mende C Hilf N Schoor O Beckhove P Bucher J. Exploiting the glioblastoma peptidome to discover novel tumour-associated antigens for immunotherapy. Brain J neurol. (2012) 135:1042–54. doi: 10.1093/brain/aws042 22418738

[158] Keskin DB Anandappa AJ Sun J Tirosh I Mathewson ND Li S. Neoantigen vaccine generates intratumoral T cell responses in phase Ib glioblastoma trial. Nature. (2019) 565:234–9. doi: 10.1038/s41586-018-0792-9 PMC6546179 30568305

[159] Papachristofilou A Hipp MM Klinkhardt U Früh M Sebastian M Weiss C. Phase Ib evaluation of a self-adjuvanted protamine formulated mRNA-based active cancer immunotherapy, BI1361849 (CV9202), combined with local radiation treatment in patients with stage IV non-small cell lung cancer. J Immunother. Cancer. (2019) 7:38. doi: 10.1186/s40425-019-0520-5 30736848 PMC6368815

[160] Rittig SM Haentschel M Weimer KJ Heine A Müller MR Brugger W. Long-term survival correlates with immunological responses in renal cell carcinoma patients treated with mRNA-based immunotherapy. Oncoimmunology. (2016) 5:e1108511. doi: 10.1080/2162402X.2015.1108511 27467913 PMC4910748

[161] Ali OA Lewin SA Dranoff G Mooney DJ. Vaccines combined with immune checkpoint antibodies promote cytotoxic T-cell activity and tumor eradication. Cancer Immunol Res. (2016) 4:95–100. doi: 10.1158/2326-6066.CIR-14-0126 26669718 PMC4740221

[162] Soares KC Rucki AA Wu AA Olino K Xiao Q Chai Y. PD-1/PD-L1 blockade together with vaccine therapy facilitates effector T-cell infiltration into pancreatic tumors. J Immunother. (2015) 38:1–11. doi: 10.1097/CJI.0000000000000062 25415283 PMC4258151

[163] Rosenblatt J Glotzbecker B Mills H Vasir B Tzachanis D Levine JD. PD-1 blockade by CT-011, anti-PD-1 antibody, enhances ex vivo T-cell responses to autologous dendritic cell/myeloma fusion vaccine. J Immunother. (2011) 34:409–18. doi: 10.1097/CJI.0b013e31821ca6ce PMC3142955 21577144

[164] Zahm CD Colluru VT McNeel DG. Vaccination with high-affinity epitopes impairs antitumor efficacy by increasing PD-1 expression on CD8(+) T cells. Cancer Immunol Res. (2017) 5:630–41. doi: 10.1158/2326-6066.CIR-16-0374 PMC5821110 28634215

[165] Zahm CD Moseman JE Delmastro LE. G Mcneel, D. et al. PD-1 and LAG-3 blockade improve antitumor vaccine efficacy. Oncoimmunology. (2021) 10:1912892. doi: 10.1080/2162402X.2021.1912892 33996265 PMC8078506

[166] Antonios JP Soto H Everson RG Orpilla J Moughon D Shin N. PD-1 blockade enhances the vaccination-induced immune response in glioma. JCI Insight. (2016) 1:e87059. doi: 10.1172/jci.insight.87059 27453950 PMC4951098

[167] Lim WA June CH. The principles of engineering immune cells to treat cancer. Cell. (2017) 168:724–40. doi: 10.1016/j.cell.2017.01.016 PMC5553442 28187291

[168] Wei J Han X Bo J Han W. Target selection for CAR-T therapy. J Hematol Oncol. (2019) 12:62. doi: 10.1186/s13045-019-0758-x 31221182 PMC6587237

[169] Brown CE Warden CD Starr R Deng X Badie B Yuan YC. Glioma IL13Ralpha2 is associated with mesenchymal signature gene expression and poor patient prognosis. PloS One. (2013) 8:e77769. doi: 10.1371/journal.pone.0077769 24204956 PMC3800130

[170] Thaci B Brown CE Binello E Werbaneth K Sampath P Sengupta S. Significance of interleukin-13 receptor alpha 2-targeted glioblastoma therapy. Neuro-Oncol. (2014) 16:1304–12. doi: 10.1093/neuonc/nou045 PMC4165413 24723564

[171] Jarboe JS Johnson KR Choi Y Lonser RR Park JK. Expression of interleukin-13 receptor alpha2 in glioblastoma multiforme: implications for targeted therapies. Cancer Res. (2007) 67:7983–6. doi: 10.1158/0008-5472.CAN-07-1493 17804706

[172] Joshi BH Puri RA Leland P Varricchio F Gupta G Kocak M. Identification of interleukin-13 receptor A2 chain overexpression in situ in high-grade diffusely infiltrative pediatric brainstem glioma. Neuro-Oncol. (2008) 10:265–74. doi: 10.1215/15228517-2007-066 PMC2563049 18430795

[173] Kawakami M Kawakami K Takahashi S Abe M Puri RK. Analysis of interleukin-13 receptor alpha2 expression in human pediatric brain tumors. Cancer. (2004) 101:1036–42. doi: 10.1002/cncr.20470 15329913

[174] Brown CE Badie B Barish ME Weng L Ostberg JR Chang WC. Bioactivity and safety of IL13Rα2-redirected chimeric antigen receptor CD8+ T cells in patients with recurrent glioblastoma. Clin Cancer Res. (2015) 21:4062–72. doi: 10.1158/1078-0432.CCR-15-0428 PMC4632968 26059190

[175] Brown CE Alizadeh D Starr R Weng L Wagner JR Naranjo A. Regression of glioblastoma after chimeric antigen receptor T-cell therapy. Engl J Med. (2016) 375:2561–9. doi: 10.1056/NEJMoa1610497 PMC5390684 28029927

[176] O'Rourke DM Nasrallah MP Desai A Melenhorst JJ Mansfield K Morrissette JJD. A single dose of peripherally infused EGFRvIII-directed CAR T cells mediates antigen loss and induces adaptive resistance in patients with recurrent glioblastoma. Sci Transl Med. (2017) 9:eaaa0984. doi: 10.1126/scitranslmed.aaa0984 28724573 PMC5762203

[177] Jiang H Gao H Kong J Song B Wang P Shi B. Selective targeting of glioblastoma with EGFRvIII/EGFR bitargeted chimeric antigen receptor T cell. Cancer Immunol Res. (2018) 6:1314–26. doi: 10.1158/2326-6066.CIR-18-0044 30201736

[178] Abbott RC Verdon DJ Gracey FM Hughes-Parry HE Iliopoulos M Watson KA. Novel high-affinity EGFRvIII-specific chimeric antigen receptor T cells effectively eliminate human glioblastoma. Clin Trans Immunol. (2021) 10:e1283. doi: 10.1002/cti2.1317 PMC8106904 33976881

[179] Abbott RC Iliopoulos M Watson KA Arcucci V Go M Hughes-Parry HE. Human EGFRvIII chimeric antigen receptor T cells demonstrate favorable safety profile and curative responses in orthotopic glioblastoma. Clin Trans Immunol. (2023) 12:e1440. doi: 10.1002/cti2.1440 PMC9986233 36890859

[180] Roskoski R. The erbB/HER family of protein-tyrosine kinases and cancer. Pharmacol Res. (2014) 79:34–74. doi: 10.1016/j.phrs.2013.11.002 24269963

[181] Iqbal N Iqbal N. Human epidermal growth factor receptor 2 (HER2) in cancers: overexpression and therapeutic implications. Mol Biol Int. (2014) 2014:1–9. doi: 10.1155/2014/852748 PMC4170925 25276427

[182] Thanindratarn P Dean DC Nelson SD Hornicek FJ Duan Z. Chimeric antigen receptor T (CAR-T) cell immunotherapy for sarcomas: From mechanisms to potential clinical applications. Cancer Treat Rev. (2020) 82:101934. doi: 10.1016/j.ctrv.2019.101934 31794912

[183] Ahmed N Salsman VS Kew Y Shaffer D Powell S Zhang YJ. HER2-specific T cells target primary glioblastoma stem cells and induce regression of autologous experimental tumors. Clin Cancer Res an Off J Am Assoc Cancer Res. (2010) 16:474–85. doi: 10.1158/1078-0432.CCR-09-1322 PMC3682507 20068073

[184] Ahmed N Brawley V Hegde M Bielamowicz K Kalra M Landi D. HER2-specific chimeric antigen receptor-modified virus-specific T cells for progressive glioblastoma: a phase 1 dose-escalation trial. JAMA Oncol. (2017) 3:1094–101. doi: 10.1001/jamaoncol.2017.0184 PMC5747970 28426845

[185] Schneider JR Kwan K Boockvar JA. Use of HER2-specific chimeric antigen receptor-modified virus-specific T cells as a potential therapeutic for progressive HER2-positive glioblastoma. Neurosurgery. (2017) 81:N42–n3. doi: 10.1093/neuros/nyx449 29088463

[186] Chow KK Naik S Kakarla S Brawley VS Shaffer DR Yi Z. T cells redirected to EphA2 for the immunotherapy of glioblastoma. Mol Ther J Am Soc Gene Ther. (2013) 21:629–37. doi: 10.1038/mt.2012.210 PMC3589173 23070117

[187] Miao H Gale NW Guo H Qian J Petty A Kaspar J. EphA2 promotes infiltrative invasion of glioma stem cells in *vivo* through cross-talk with Akt and regulates stem cell properties. Oncogene. (2015) 34:558–67. doi: 10.1038/onc.2013.590 PMC4119862 24488013

[188] Gai QJ Fu Z He J Mao M Yao XX Qin Y. EPHA2 mediates PDGFA activity and functions together with PDGFRA as prognostic marker and therapeutic target in glioblastoma. Signal transduct. targeted Ther. (2022) 7:33. doi: 10.1038/s41392-021-00855-2 PMC8807725 35105853

[189] An Z Hu Y Bai Y Zhang C Xu C Kang X. Antitumor activity of the third generation EphA2 CAR-T cells against glioblastoma is associated with interferon gamma induced PD-L1. Oncoimmunology. (2021) 10:1960728. doi: 10.1080/2162402X.2021.1960728 34408922 PMC8366541

[190] Bielamowicz K Fousek K Byrd TT Samaha H Mukherjee M Aware N. Trivalent CAR T cells overcome interpatient antigenic variability in glioblastoma. Neuro-oncology. (2018) 20:506–18. doi: 10.1093/neuonc/nox182 PMC5909636 29016929

[191] Hegde M Corder A Chow KK Mukherjee M Ashoori A Kew Y. Combinational targeting offsets antigen escape and enhances effector functions of adoptively transferred T cells in glioblastoma. Mol Ther J Am Soc Gene Ther. (2013) 21:2087–101. doi: 10.1038/mt.2013.185 PMC3831041 23939024

[192] Hegde M Mukherjee M Grada Z Pignata A Landi D Navai SA. Tandem CAR T cells targeting HER2 and IL13Rα2 mitigate tumor antigen escape. J Clin Invest. (2016) 126:3036–52. doi: 10.1172/JCI83416 PMC4966331 27427982

[193] Schmidts A Srivastava AA Ramapriyan R Bailey SR Bouffard AA Cahill DP. Tandem chimeric antigen receptor (CAR) T cells targeting EGFRvIII and IL-13Rα2 are effective against heterogeneous glioblastoma. Neuro-oncol adv. (2023) 5:vdac185. doi: 10.1093/noajnl/vdac185 PMC9896600 36751672

[194] Mount CW Majzner RG Sundaresh S Arnold EP Kadapakkam M Haile S. Potent antitumor efficacy of anti-GD2 CAR T cells in H3-K27M(+) diffuse midline gliomas. Nat Med. (2018) 24:572–9. doi: 10.1038/s41591-018-0006-x PMC6214371 29662203

[195] Majzner RG Ramakrishna S Yeom KW Patel S Chinnasamy H Schultz LM. GD2-CAR T cell therapy for H3K27M-mutated diffuse midline gliomas. Nature. (2022) 603:934–41. doi: 10.1038/s41586-022-04489-4 PMC8967714 35130560

[196] Nehama D Di Ianni N Musio S Du H Patané M Pollo B. B7-H3-redirected chimeric antigen receptor T cells target glioblastoma and neurospheres. EBioMedicine. (2019) 47:33–43. doi: 10.1016/j.ebiom.2019.08.030 31466914 PMC6796553

[197] Majzner RG Theruvath JL Nellan A Heitzeneder S Cui Y Mount CW. CAR T cells targeting B7-H3, a pan-cancer antigen, demonstrate potent preclinical activity against pediatric solid tumors and brain tumors. Clin Cancer Res an Off J Am Assoc Cancer Res. (2019) 25:2560–74. doi: 10.1158/1078-0432.CCR-18-0432 PMC8456711 30655315

[198] Wang D Starr R Chang WC Aguilar B Alizadeh D Wright SL. Chlorotoxin-directed CAR T cells for specific and effective targeting of glioblastoma. Sci Trans Med. (2020) 12:eaaw2672. doi: 10.1126/scitranslmed.aaw2672 PMC7500824 32132216

[199] Hänsch L Peipp M Mastall M Villars D Myburgh R Silginer M. Chimeric antigen receptor (CAR) T cell-based targeting of CD317 as a novel immunotherapeutic strategy against glioblastoma. Neuro-oncology. (2023) 25:2001–14. doi: 10.1093/neuonc/noad108 PMC10628943 37335916

[200] Qian B-Z Pollard JW. Macrophage diversity enhances tumor progression and metastasis. Cell. (2010) 141:39–51. doi: 10.1016/j.cell.2010.03.014 20371344 PMC4994190

[201] Grégoire H Roncali L Rousseau A Chérel M Delneste Y Jeannin P. Targeting tumor associated macrophages to overcome conventional treatment resistance in glioblastoma. Front Pharmacol. (2020) 11:368. doi: 10.3389/fphar.2020.00368 32322199 PMC7158850

[202] Stanley ER Chitu V. CSF-1 receptor signaling in myeloid cells. Cold Spring Harb Perspect Biol. (2014) 6:a021857. doi: 10.1101/cshperspect.a021857 24890514 PMC4031967

[203] Butowski N Colman H De Groot JF Omuro AM Nayak L Wen PY. Orally administered colony stimulating factor 1 receptor inhibitor PLX3397 in recurrent glioblastoma: an Ivy Foundation Early Phase Clinical Trials Consortium phase II study. Neuro Oncol. (2016) 18:557–64. doi: 10.1093/neuonc/nov245 PMC4799682 26449250

[204] Brown EJ Frazier WA. Integrin-associated protein (CD47) and its ligands. Trends Cell Biol. (2001) 11:130–5. doi: 10.1016/S0962-8924(00)01906-1 11306274

[205] Jaiswal S Jamieson CHM Pang WW Park CY Chao MP Majeti R. CD47 is upregulated on circulating hematopoietic stem cells and leukemia cells to avoid phagocytosis. Cell. (2009) 138:271–85. doi: 10.1016/j.cell.2009.05.046 PMC2775564 19632178

[206] Hutter G Theruvath J Graef CM Zhang M Schoen MK Manz EM. Microglia are effector cells of CD47-SIRPα antiphagocytic axis disruption against glioblastoma. Proc Natl Acad Sci U S A. (2019) 116:997–1006. doi: 10.1073/pnas.1721434116 30602457 PMC6338872

[207] Hsu SPC Chen Y-C Chiang H-C Huang Y-C Huang C-C Wang H-E. Rapamycin and hydroxychloroquine combination alters macrophage polarization and sensitizes glioblastoma to immune checkpoint inhibitors. J Neurooncol. (2020) 146:417–26. doi: 10.1007/s11060-019-03360-3 PMC7000510 32020472

